# Efficacy and Safety of *Syzygium cumini* and Related Myrtaceae Interventions for Dysglycemia: A Systematic Review and Meta-Analysis of Randomized Controlled Trials

**DOI:** 10.3390/foods15132332

**Published:** 2026-07-01

**Authors:** Thitinat Duangchan, Kunanya Kuakul, Cholthicha Saipin, Hanna Pongyeela, Hazna Sarana, Kittikorn Wangriatisak, Moragot Chatatikun, Atthaphong Phongphithakchai

**Affiliations:** 1 School of Allied Health Sciences, Walailak University, Nakhon Si Thammarat 80160, Thailand; thitinat.du@wu.ac.th (T.D.); kunanya.ku@mail.wu.ac.th (K.K.); cholthicha.sa@mail.wu.ac.th (C.S.); hanna.po@mail.wu.ac.th (H.P.); hazna.sa@mail.wu.ac.th (H.S.); moragot.ch@wu.ac.th (M.C.); 2 Hematology and Transfusion Science Research Center (HTSRC), Walailak University, Nakhon Si Thammarat 80160, Thailand; 3 Division of Rheumatology, Department of Medicine Solna, Karolinska Institutet, Karolinska University Hospital, 171 76 Stockholm, Sweden; kittikorn.wangriatisak@ki.se; 4 Center for Molecular Medicine, Karolinska Institutet, 171 76 Stockholm, Sweden; 5 Research Excellence Center for Innovation and Health Products (RECIHP), Walailak University, Nakhon Si Thammarat 80160, Thailand; 6 Nephrology Unit, Division of Internal Medicine, Faculty of Medicine, Prince of Songkla University, Songkhla 90110, Thailand

**Keywords:** *Syzygium cumini*, jamun, Myrtaceae, type 2 diabetes, HbA1c, fasting glucose, systematic review, meta-analysis

## Abstract

**Background**: *Syzygium cumini* and other Myrtaceae plants are widely used as traditional remedies for dysglycemia, yet clinical evidence remains fragmented and heterogeneous. This study aimed to evaluate the efficacy and safety of *S. cumini* and related Myrtaceae interventions in individuals with dysglycemia. **Methods**: A systematic review and meta-analysis of randomized controlled trials was conducted in accordance with PRISMA 2020 and registered in PROSPERO (CRD420261332539). PubMed, Scopus, Embase, MEDLINE, and Web of Science were searched from inception to 3 September 2025. Eligible trials enrolled participants with type 2 diabetes mellitus (T2DM) or prediabetes and evaluated Myrtaceae interventions versus controls. Random-effects meta-analyses were performed to estimate mean differences (MDs) with 95% confidence intervals (CIs). **Results**: Thirteen trials comprising 802 participants were included. Myrtaceae interventions were associated with a statistically significant but modest reduction in fasting plasma glucose (MD −14.40 mg/dL, 95% CI −23.12 to −5.67; *I*^2^ = 98.57%). However, effects on postprandial glucose (MD −12.99 mg/dL, 95% CI −27.74 to 1.76; *I*^2^ = 97.93%) and HbA1c (MD −0.46%, 95% CI −0.98 to 0.06; *I*^2^ = 99.20%) were not statistically significant. Overall effects on lipid outcomes and laboratory safety markers were also not significant. Subgroup analyses suggested possible variation by participant type, plant part, formulation composition, comparator type, and treatment duration, but these findings were exploratory and accompanied by substantial heterogeneity. **Conclusions**: Myrtaceae interventions may provide a modest short-term reduction in fasting glycemia among adults with T2DM or prediabetes. However, the clinical significance and generalizability of this finding remain uncertain due to high heterogeneity, short follow-up, absence of low-risk trials, and low to very low certainty of evidence. Current evidence does not support consistent benefits for PPG, HbA1c, lipid outcomes, or long-term safety. These interventions should be considered promising but unproven adjuncts rather than alternatives to standard dysglycemia management.

## 1. Introduction

Dysglycemia is best understood as a continuum of abnormal glucose regulation that spans impaired fasting glucose (IFG), impaired glucose tolerance (IGT), and overt type 2 diabetes mellitus (T2DM). It commonly coexists with insulin resistance, obesity, and metabolic syndrome [1]. The global prevalence of dysglycemia has reached alarming proportions, posing a formidable challenge to public health systems. In 2021, an estimated 536.6 million adults aged 20–79 years were living with diabetes worldwide, and this number is projected to rise to 783.2 million by 2045. In the same year, global prediabetes prevalence was estimated at 5.8% for IFG and 9.1% for IGT [2]. Importantly, prediabetes is associated with increased risk of progression to T2DM, cardiovascular disease, and premature mortality, and vascular injury may begin before diagnostic thresholds for overt diabetes are crossed [3,4]. Despite the availability of potent pharmacological agents, long-term glycemic management remains suboptimal for a significant proportion of patients. This difficulty is driven not only by the progressive nature of insulin resistance and β-cell dysfunction but also by therapeutic inertia in routine practice and intolerance to commonly used agents [5]. Therapeutic inertia remains a well-recognized barrier in T2DM management, while even first-line metformin is frequently limited by gastrointestinal adverse effects such as diarrhea, nausea, and abdominal discomfort [6]. Consequently, there is a burgeoning interest in adjunctive dietary interventions and phytotherapeutic agents that may provide multi-target glycemic support with acceptable tolerability [7].

Among the diverse botanical families investigated for metabolic health, Myrtaceae is of particular interest because several of its species have longstanding ethnobotanical use for glycemic control and share broadly overlapping polyphenol-rich phytochemical profiles. In the clinical trial literature relevant to dysglycemia, *Syzygium cumini* (synonym *Eugenia jambolana*, commonly known as jamun or Java plum) is the most extensively studied species, particularly its seed, fruit, and leaf preparations, which have been used in Ayurvedic and other traditional medicine systems for centuries [8]. Interest in this family has also extended to other traditionally used Myrtaceae species, including *Psidium guajava* (guava), *Syzygium aromaticum* (clove), *Syzygium polyanthum* (Indonesian bay leaf or salam leaf), *Plinia jaboticaba* (jabuticaba), and *Acca sellowiana* (feijoa), which contain bioactive secondary metabolites that may plausibly influence glucose homeostasis [9,10,11]. Considering these species together provides an opportunity to examine whether related Myrtaceae interventions, despite differences in plant part and preparation, show consistent clinical signals across randomized trials. At the same time, such synthesis requires cautious interpretation, as differences among species, formulations, and phytochemical standardization may lead to preparation-specific rather than uniform family-level effects.

The biological rationale for investigating Myrtaceae interventions in glycemic homeostasis is largely derived from their polyphenol-rich composition, particularly ellagitannins, gallic acid, anthocyanins, and flavonoids [12]. Experimental and in vitro studies suggest that these compounds may attenuate carbohydrate digestion and intestinal glucose absorption, modulate postprandial glucose excursions, improve peripheral glucose uptake, and enhance insulin sensitivity through pathways that include inhibition of digestive enzymes and regulation of glucose transport and cellular signaling [13,14]. Such mechanisms are relevant to dysglycemia because insulin resistance and metabolic syndrome are closely linked to chronic low-grade inflammation and oxidative stress [15]. However, mechanistic plausibility alone does not establish clinical efficacy. Effects observed in preclinical models may not translate consistently to humans because of differences in dose, bioavailability, formulation, baseline metabolic status, and treatment duration.

Consistent with this uncertainty, human clinical trial evidence for Myrtaceae interventions remains characterized by significant inconsistencies and high inter-study heterogeneity. Early randomized studies of *S. cumini* leaf preparations failed to demonstrate meaningful antihyperglycemic effects and did not show a benefit over placebo or glyburide in adults with T2DM [16,17]. In contrast, later randomized trials involving other Myrtaceae interventions reported improved intestinal glucose handling with guava fruit extract in healthy adults and reduced postprandial glycemia and inflammation with jaboticaba peel in adults with metabolic syndrome [18,19]. These discrepancies are likely attributable to several factors, including variations in the plant parts used, differences in preparation, and diversity in the study populations, ranging from healthy volunteers to long-standing diabetics. Furthermore, trials often utilize small sample sizes and short durations, which may be insufficient to observe changes in glycated hemoglobin (HbA1c). The use of diverse comparators, ranging from inert placebos to active agents like metformin, further complicates the interpretation of their relative therapeutic value.

Previous botanical and nutraceutical meta-analyses have commonly pooled related plant-based interventions when they share traditional use, botanical relatedness, or major bioactive phytochemical classes, while addressing expected heterogeneity through subgroup and sensitivity analyses [20,21,22,23]. For example, cinnamon meta-analyses have synthesized trials of cinnamon products despite potential differences or incomplete reporting of *Cinnamomum* species, plant parts, and preparations [20,21]. Similarly, green tea and catechin meta-analyses have combined trials using tea beverages, extracts, and catechin-based preparations because these interventions share a core phytochemical basis, while acknowledging variation in formulation and dose [22,23]. This precedent supports an exploratory synthesis of related Myrtaceae interventions, provided that pooled estimates are interpreted cautiously and potential sources of heterogeneity are examined.

Therefore, a rigorous systematic review and meta-analysis of randomized controlled trials is warranted to clarify the efficacy and safety of *S. cumini* and related Myrtaceae interventions in individuals with dysglycemia. In the present review, eligible Myrtaceae interventions were combined to estimate an exploratory overall clinical signal, rather than to assume a uniform family-level effect. We also examined whether clinical effects differed by species, plant part, formulation composition, comparator type, and treatment duration. Key outcomes included fasting plasma glucose, postprandial glucose, and glycated hemoglobin. Secondary cardiometabolic outcomes and safety markers included lipid parameters, liver enzymes, and serum creatinine, providing a more clinically relevant synthesis of the available evidence.

## 2. Materials and Methods

### 2.1. Protocol Registration

This systematic review and meta-analysis was conducted and reported in accordance with the Preferred Reporting Items for Systematic Reviews and Meta-Analyses (PRISMA) 2020 statement. The review protocol was prospectively registered in the International Prospective Register of Systematic Reviews (PROSPERO) under the registration number CRD420261332539.

### 2.2. Search Strategy

A comprehensive electronic search strategy was developed and applied to PubMed, Scopus, Embase, MEDLINE, and Web of Science from inception to 3 September 2025. No publication date limits were applied, and searches were not restricted by language at the search stage. The strategies combined controlled vocabulary (e.g., MeSH and Emtree terms) with free-text keywords using Boolean operators. The main concepts were: (1) *Syzygium cumini* and related Myrtaceae terms (including synonyms and common names such as *Eugenia jambolana*, “jamun/Java plum,” and broader Myrtaceae genus/family terms) and (2) dysglycemia-related terms (e.g., diabetes, prediabetes, hyperglyc*, insulin resist*, metabolic syndrome). The summary of database-specific search strategies is presented in Table 1, and the full search strategies are provided in Supplementary Table S1.

### 2.3. Study Selection and Eligibility Criteria

All retrieved records were imported into Rayyan (Rayyan Systems Inc., Cambridge, MA, USA), and duplicates were removed. Four reviewers (K.K., H.P., H.S., and C.S.) independently screened studies in two stages: (1) title/abstract screening and (2) full-text assessment. Discrepancies were resolved through discussion and adjudication by another reviewer (T.D.). Eligibility criteria were prespecified using the PICOS framework. The population of interest was adults with dysglycemia, defined as type 2 diabetes mellitus (T2DM) or prediabetes, including impaired fasting glucose or impaired glucose tolerance. Trials conducted exclusively in healthy, normoglycemic, non-diabetic, or metabolic syndrome populations without explicit eligibility for T2DM or prediabetes were excluded. Eligible interventions were oral preparations derived from Myrtaceae plants, regardless of plant part, formulation, dose, or treatment duration. Interventions could be administered as monotherapy or an add-on to stable usual care. Both single-botanical Myrtaceae preparations and multi-ingredient formulations containing an explicitly stated Myrtaceae component were eligible. Homeopathic preparations were included only if they met all eligibility criteria as randomized controlled trials of oral Myrtaceae-derived interventions reporting relevant glycemic or safety outcomes. Eligible comparators included placebo, usual care, active comparators, food controls, or other appropriate control conditions. Studies were required to report at least one extractable quantitative outcome related to glycemic control, lipid profile, or laboratory safety markers. We excluded non-randomized studies, animal studies, in vitro studies, case reports or case series, reviews, and trials in which the Myrtaceae intervention could not be clearly identified.

### 2.4. Data Extraction

Four reviewers (K.K., H.P., H.S., and C.S.) independently extracted data using a standardized Google Sheets extraction form (Google LLC, Mountain View, CA, USA). Disagreements were resolved through discussion and adjudication by another reviewer (T.D.). Extracted information included study characteristics (title, first author, publication year, country, and study design), participant characteristics (population group, diagnostic criteria, sample size, mean age, and sex distribution), and intervention details (Myrtaceae species, plant part, preparation/formulation, dose, and intervention duration). Extracted primary outcomes were fasting plasma glucose (FPG), postprandial glucose (PPG), and HbA1c. Secondary outcomes included lipid profile (total cholesterol, triglycerides, and HDL) and safety markers (AST, ALT, and creatinine). When data were presented only graphically, numeric values were digitized using PlotDigitizer software 3.1.6 (https://plotdigitizer.com/, accessed on 1 January 2026). Units were harmonized prior to analysis (glucose converted using 1 mmol/L = 18 mg/dL; HbA1c analyzed as percentage).

### 2.5. Risk-of-Bias Assessment

Risk of bias in the included randomized trials was assessed using the Cochrane Risk of Bias 2 (RoB 2) tool. Two reviewers (M.C. and T.D.) independently evaluated each study across the five RoB 2 domains, including randomization process, deviations from intended interventions, missing outcome data, outcome measurement, and selection of the reported result. Each domain was judged as low risk of bias, some concerns, or high risk of bias, and an overall judgment was assigned for each study. Any disagreements were resolved by discussion and consensus.

### 2.6. Data Synthesis and Statistical Analysis

Meta-analyses were performed using random-effects models with restricted maximum likelihood (REML) estimation. The primary meta-analysis pooled eligible Myrtaceae interventions to estimate the overall average effect across related botanical interventions. Continuous outcomes were summarized as mean differences (MDs) with 95% confidence intervals (CIs) using post-intervention values. MDs were selected because each outcome was pooled separately in the same clinical unit after unit harmonization. Statistical heterogeneity was assessed using *I*^2^ and interpreted as low, moderate, or high at approximately 25%, 50%, and 75%, respectively. Robustness of pooled estimates was evaluated using leave-one-out sensitivity analyses. Prespecified subgroup analyses were conducted by participant phenotype (T2DM vs. prediabetes), plant species (*S. cumini* vs. other Myrtaceae), plant part, formulation composition (single-botanical vs. mixed/polyherbal), comparator type (placebo/vehicle vs. metformin vs. others), treatment duration categories (0–8, 9–16, 17–24 weeks), and publication period. Random-effects meta-regression (REML) was conducted using treatment duration (weeks) and publication year as continuous moderators. Publication bias and small-study effects were assessed by visual inspection of funnel plots and Egger’s regression test. All analyses were performed in Stata version 19 (StataCorp., College Station, TX, USA), with *p* < 0.05 considered statistically significant.

### 2.7. Certainty of Evidence Assessment

The certainty of evidence for outcomes, including FPG, PPG, HbA1c, total cholesterol, triglycerides, HDL, AST, ALT, and creatinine, was assessed using the Grading of Recommendations Assessment, Development and Evaluation (GRADE) approach. As all included studies were randomized controlled trials, the certainty of evidence was initially rated as high and was downgraded for risk of bias, inconsistency, indirectness, imprecision, and publication bias. Risk of bias was informed by RoB 2 judgments; inconsistency was assessed using heterogeneity, direction of effects, sensitivity analyses, subgroup analyses, and meta-regression; indirectness was considered in relation to the review question, population, interventions, comparators, and outcomes; imprecision was judged based on confidence interval width, crossing of the line of no effect, and sample size; and publication bias was assessed using funnel plots and Egger’s test. Certainty was rated as high, moderate, low, or very low.

## 3. Results

### 3.1. Study Selection

**Figure 1:**
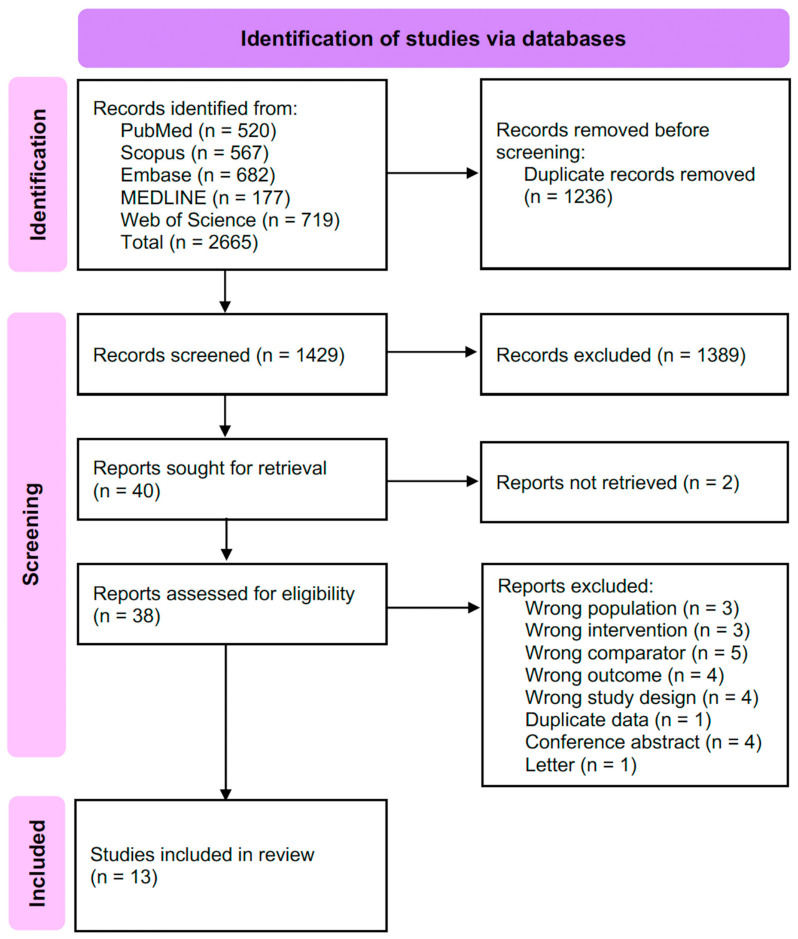
PRISMA 2020 flow diagram of study selection.

A total of 2665 records were identified from database searches (PubMed; *n* = 520, Scopus; *n* = 567, Embase; *n* = 682, MEDLINE; *n* = 177, and Web of Science; *n* = 719). After removing 1236 duplicates, 1429 records were screened by title and abstract, of which 1389 were excluded. We sought 40 full-text reports for retrieval; 2 could not be retrieved, leaving 38 reports assessed for eligibility. Of these, 25 were excluded (wrong population; *n* = 3, wrong intervention; *n* = 3, wrong comparator; *n* = 5, wrong outcome; *n* = 4, wrong study design; *n* = 4, duplicate data; *n* = 1, conference abstracts; *n* = 4, and letter *n* = 1). Overall, 13 studies [16,24,25,26,27,28,29,30,31,32,33,34,35] met the inclusion criteria and were included in the systematic review (Figure 1).

### 3.2. Characteristics of Included Studies

Thirteen RCTs involving 802 participants were included. The studies were published between 2001 and 2025 and were conducted predominantly in India (*n* = 9), with additional trials from Indonesia (*n* = 2), Brazil (*n* = 1), and New Zealand (*n* = 1). Sample sizes ranged from 12 to 120 participants. All trials enrolled adults with dysglycemia, including T2DM or prediabetes. Eight trials enrolled participants with T2DM (432 participants), four enrolled participants with prediabetes (301 participants), and one trial reported by Majeed et al. (2021) included two independent cohorts, which were handled as separate comparisons in the meta-analysis (40 T2DM and 29 prediabetes participants) [27]. All included studies used a parallel-group design. Blinding varied across trials, with several described as double-blind, others as single-blind, and one as an open-label study. Intervention duration ranged from 2 to 24 weeks.

The included interventions were clinically diverse and represented an exploratory class-level synthesis of related Myrtaceae-based interventions rather than a single uniform product effect. *Syzygium cumini* was the most frequently evaluated species (9 trials). Other Myrtaceae interventions included *Acca sellowiana* (1 trial), *Syzygium aromaticum* (1 trial), and *Syzygium polyanthum* (2 trials). Plant parts varied across trials and included seed, seed kernel, leaf, fruit, and bud. Formulations included powders, capsules, tablets, tea, tinctures, and homeopathic preparations.

Intervention composition also differed across studies. Some trials evaluated single-botanical Myrtaceae preparations (7 trials), whereas others evaluated multi-ingredient or polyherbal formulations in which a Myrtaceae species was explicitly stated as a component (6 trials). Therefore, effects observed in mixed formulations cannot be attributed solely to the Myrtaceae component. Comparator types were also heterogeneous, including placebo, metformin, food controls, and other active or pragmatic control conditions. These differences in species, plant part, formulation, dose, duration, and comparator type were considered when interpreting the pooled estimates and were further explored through subgroup analyses (Table 2).

### 3.3. Risk of Bias

**Figure 2:**
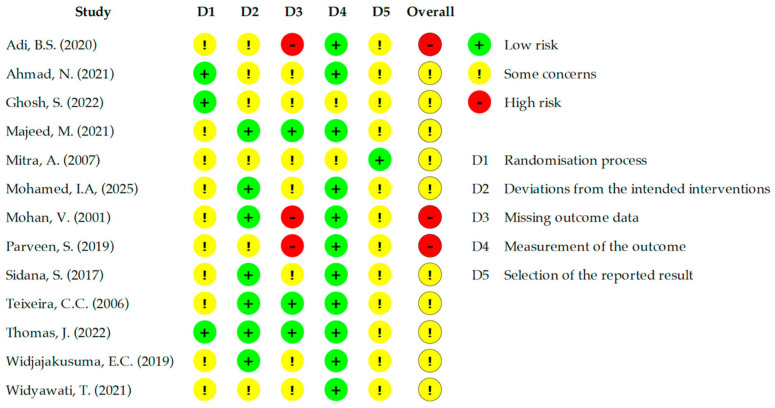
Risk-of-bias assessment of included randomized controlled trials using RoB 2 [16,24,25,26,27,28,29,30,31,32,33,34,35].

Using the RoB 2 assessment criteria, 10 trials were judged to have some concerns, and 3 were judged to be at high risk of bias overall. No included trial met criteria for an overall low risk of bias. This absence of low-risk trials represents a major limitation of the evidence base and was considered when interpreting the pooled findings. The high-risk judgments were primarily driven by missing outcome data related to glycemic status [20,30,31]. Among trials judged as having some concerns, the most frequent issues were incomplete reporting of the randomization process or allocation concealment, and potential selective reporting, commonly due to limited access to prespecified analysis plans or insufficient reporting to verify consistency across outcomes and time points. Concerns regarding deviations from intended interventions were mainly related to open-label or single-blind designs, uncertainty about masking success, or pragmatic interventions. By contrast, measurement of outcomes was generally judged low risk, as the main endpoints were objective laboratory measures (Figure 2).

### 3.4. Effects on Glycemic Control

#### 3.4.1. Fasting Plasma Glucose (FPG)

**Figure 3:**
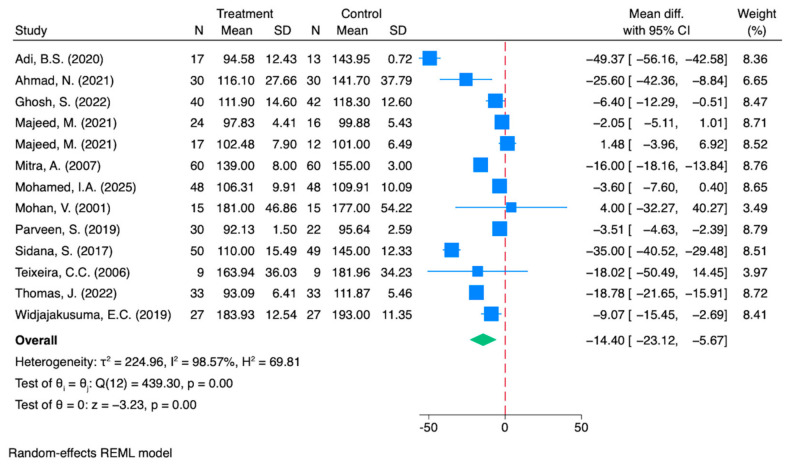
Forest plot of Myrtaceae interventions versus control for FPG (mg/dL). Random-effects meta-analyses were performed using REML. Pooled effects are expressed as MD with 95% CI [16,24,25,26,27,28,29,30,31,32,33,34]. Blue squares, individual study MDs, with square size proportional to study weight; horizontal lines, 95% CIs; green diamond, pooled MD; red dashed vertical line, line of no effect.

Thirteen RCTs contributed data on FPG, including 400 participants in the Myrtaceae intervention groups and 376 participants in the control groups. Random-effects meta-analysis (REML) showed that Myrtaceae interventions were associated with a statistically significant but modest reduction in FPG compared with controls (MD −14.40 mg/dL, 95% CI −23.12 to −5.67, *p* = 0.001). Between-study heterogeneity was considerable (*I*^2^ = 98.57%), indicating that the magnitude of the pooled estimate should not be generalized across all species, formulations, comparators, or participant groups (Figure 3). Leave-one-out sensitivity analysis showed that the direction of effect remained consistently favorable and statistically significant after sequential omission of individual studies (Supplementary Figure S1). Nevertheless, given the extreme heterogeneity, the pooled estimate should be interpreted as a preliminary average effect across diverse Myrtaceae-based interventions rather than as evidence of a uniform effect for any specific preparation.

#### 3.4.2. Postprandial Glucose (PPG)

**Figure 4:**
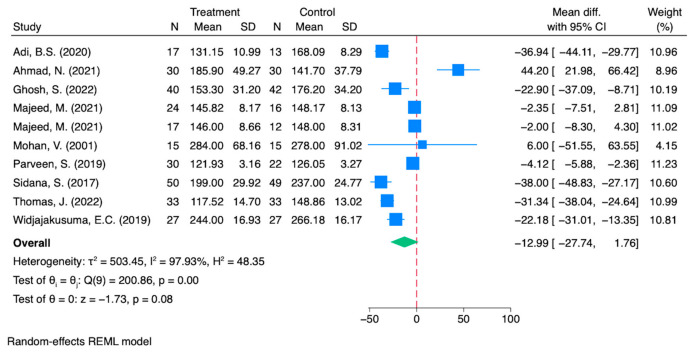
Forest plot of Myrtaceae interventions versus control for PPG (mg/dL). Random-effects meta-analyses were performed using REML. Pooled effects are expressed as MD with 95% CI [24,25,26,27,30,31,32,33,34]. Blue squares, individual study MDs, with square size proportional to study weight; horizontal lines, 95% CIs; green diamond, pooled MD; red dashed vertical line, line of no effect.

Ten studies reported PPG, comprising 283 participants in the Myrtaceae intervention groups and 259 participants in the control groups. Pooled analysis showed a non-statistically significant reduction in PPG in favor of Myrtaceae interventions compared with controls (MD −12.99 mg/dL, 95% CI −27.74 to 1.76, *p* = 0.084). Between-study heterogeneity was substantial (*I*^2^ = 97.93%) (Figure 4). Leave-one-out analyses indicated that the direction of effect remained consistently toward lower PPG after sequential omission of individual studies. However, the pooled estimate remained statistically non-significant in most iterations, except when Ahmad et al. (2021) [25] was omitted, which yielded a significant reduction in PPG (MD −18.88 mg/dL, 95% CI −29.46 to −8.30; *p* < 0.001) (Supplementary Figure S2). These findings suggest a directionally favorable but statistically unstable PPG signal, with the conclusion sensitive to study composition rather than indicating a robust class-level effect.

#### 3.4.3. HbA1c

**Figure 5:**
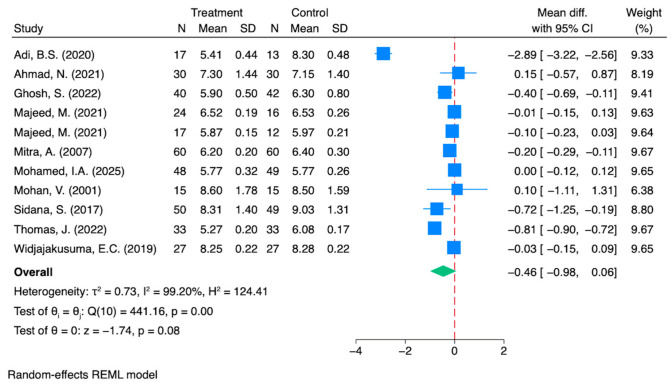
Forest plot of Myrtaceae interventions versus control for HbA1c (%). Random-effects meta-analyses were performed using REML. Pooled effects are expressed as MD with 95% CI [24,25,26,27,28,29,30,32,33,34]. Blue squares, individual study MDs, with square size proportional to study weight; horizontal lines, 95% CIs; green diamond, pooled MD; red dashed vertical line, line of no effect.

Eleven RCTs contributed HbA1c data, including 361 participants in the Myrtaceae intervention groups and 346 participants in the control groups. Overall, Myrtaceae interventions were not associated with a statistically significant improvement in HbA1c (MD −0.46%, 95% CI −0.98 to 0.06; *p* = 0.082). Heterogeneity was very high (*I*^2^ = 99.20%) (Figure 5). Leave-one-out sensitivity analysis showed that the pooled estimates generally remained directionally favorable but statistically non-significant after sequential omission of individual studies. However, omission of Adi et al. (2020) [24] resulted in a statistically significant HbA1c reduction (MD −0.23%, 95% CI −0.43 to −0.02; *p* = 0.031) (Supplementary Figure S3).

### 3.5. Subgroup Analyses of Glycemic Outcomes

#### 3.5.1. Types of Participants

**Figure 6:**
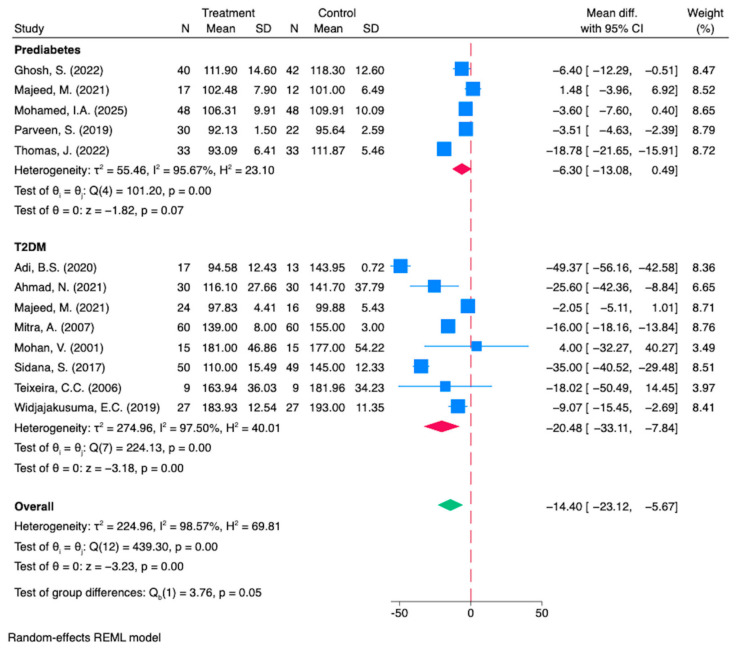
Subgroup forest plot analysis for FPG stratified by types of participants. Random-effects meta-analyses were performed using REML. Pooled effects are expressed as MD with 95% CI [16,24,25,26,27,28,29,30,31,32,33,34]. Blue squares, individual study MDs, with square size proportional to study weight; horizontal lines, 95% CIs; red diamonds, subgroup pooled MDs; green diamond, pooled MD; red dashed vertical line, line of no effect.

For FPG, the largest reduction was observed among participants with T2DM (MD −20.48 mg/dL, 95% CI −33.11 to −7.84; *I*^2^ = 97.50%), whereas the estimate in prediabetes was smaller and not statistically significant (MD −6.30 mg/dL, 95% CI −13.08 to 0.49; *I*^2^ = 95.67%) (Figure 6). For PPG, a significant reduction was observed in prediabetes (MD −14.59 mg/dL, 95% CI −28.96 to −0.22; *I*^2^ = 95.99%), whereas the estimate in T2DM was not statistically significant (MD −10.44 mg/dL, 95% CI −35.94 to 15.05; *I*^2^ = 97.46%). For HbA1c, no significant reduction was observed in either subgroup (Supplementary Table S2). These participant-stratified findings suggest possible differences in short-term glycemic response between T2DM and prediabetes, but the high heterogeneity within both strata limits firm conclusions.

#### 3.5.2. Species, Plant Part, and Composition

**Figure 7:**
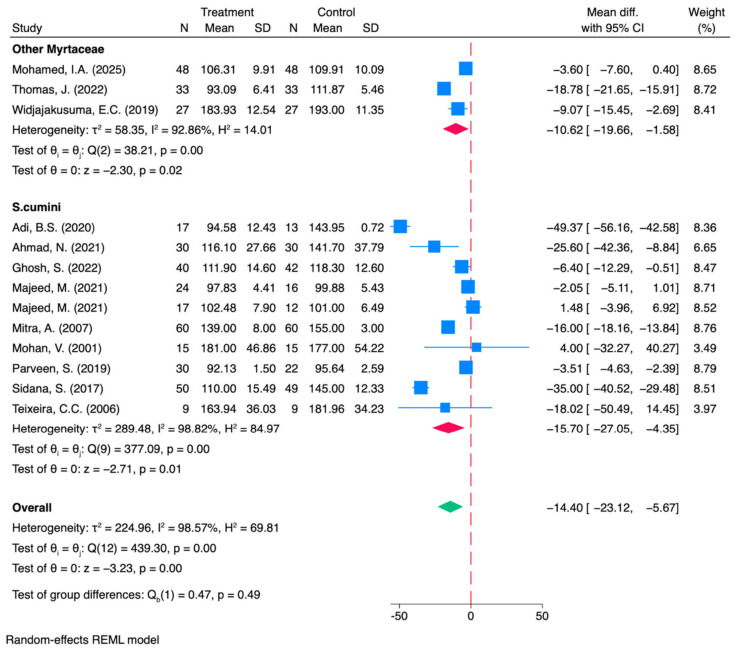
Subgroup forest plot analysis for FPG stratified by plant species. Random-effects meta-analyses were performed using REML. Pooled effects are expressed as MD with 95% CI [16,24,25,26,27,28,29,30,31,32,33,34]. Blue squares, individual study MDs, with square size proportional to study weight; horizontal lines, 95% CIs; red diamonds, subgroup pooled MDs; green diamond, pooled MD; red dashed vertical line, line of no effect.

By species, both *S. cumini* and other Myrtaceae interventions showed significant reductions in FPG. The reduction was larger in the *S. cumini* subgroup (MD −15.70 mg/dL, 95% CI −27.05 to −4.35; *I*^2^ = 98.82%) than in the other Myrtaceae subgroup (MD −10.62 mg/dL, 95% CI −19.66 to −1.58; *I*^2^ = 92.86%) (Figure 7). For PPG, other Myrtaceae interventions showed a clearer reduction (MD −27.23 mg/dL, 95% CI −36.16 to −18.30) with comparatively lower heterogeneity (*I*^2^ = 43.03%), while *S. cumini* did not show a significant effect on PPG reduction (MD −8.89 mg/dL, 95% CI −27.14 to 9.36; *I*^2^ = 98.25%). HbA1c did not differ significantly by species subgroup (Supplementary Table S2).

By plant part, seed-based interventions were associated with larger FPG reductions (MD −20.92 mg/dL, 95% CI −40.48 to −1.36; *I*^2^ = 97.36%), while leaf-based interventions also reduced FPG (MD −9.40 mg/dL, 95% CI −15.66 to −3.14; *I*^2^ = 0.00%). Fruit-based interventions demonstrated little evidence of benefit for FPG (MD −1.94 mg/dL, 95% CI −4.16 to 0.28; *I*^2^ = 0.00%) or PPG (MD −2.21 mg/dL, 95% CI −6.20 to 1.78; *I*^2^ = 0.00%). Between-subgroup differences by plant part were significant for both FPG and PPG, suggesting that plant part may contribute to variation in glycemic effects. HbA1c subgroup estimates by plant part were not statistically significant (Supplementary Table S2). However, these findings should be interpreted cautiously because some strata contained few studies and heterogeneity remained high, particularly for seed-based interventions.

When stratified by formulation composition, both mixed formulations (MD −7.83 mg/dL, 95% CI −14.16 to −1.51; *I*^2^ = 89.98%) and single-botanical interventions (MD −21.47 mg/dL, 95% CI −36.93 to −6.01; *I*^2^ = 99.06%) were associated with reduced FPG, with a larger point estimate in the single-botanical subgroup. However, the between-subgroup difference was not significant. For PPG, single-botanical interventions showed a significant reduction (MD −27.16 mg/dL, 95% CI −43.20 to −11.12; *I*^2^ = 96.74%), whereas mixed formulations showed no clear effect (MD −1.76 mg/dL, 95% CI −21.60 to 18.08; *I*^2^ = 95.66%). For HbA1c, a small but statistically significant reduction was observed in mixed formulations (MD −0.11%, 95% CI −0.21 to −0.02; *I*^2^ = 50.86%), whereas the single-botanical subgroup was not statistically significant (Supplementary Table S2).

#### 3.5.3. Types of Comparators

**Figure 8:**
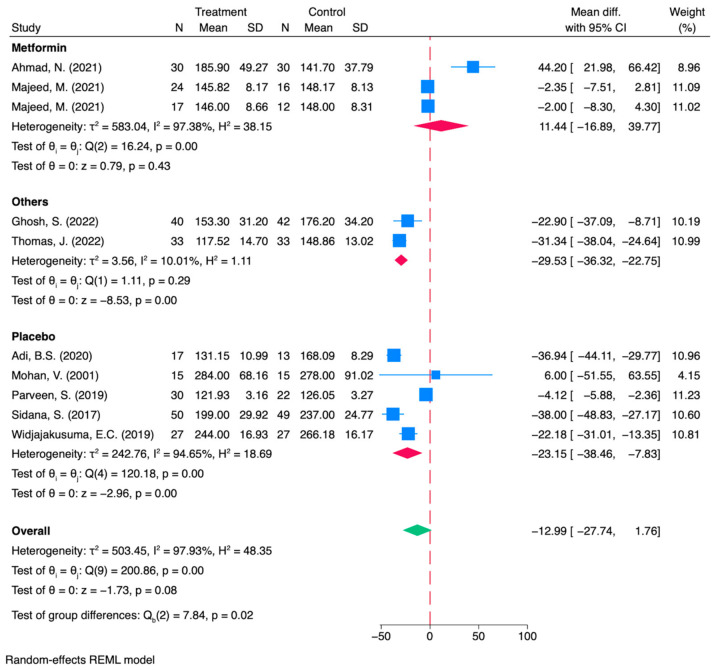
Subgroup forest plot analysis for PPG stratified by types of comparators. Random-effects meta-analyses were performed using REML. Pooled effects are expressed as MD with 95% CI [24,25,26,27,30,31,32,33,34]. Blue squares, individual study MDs, with square size proportional to study weight; horizontal lines, 95% CIs; red diamonds, subgroup pooled MDs; green diamond, pooled MD; red dashed vertical line, line of no effect.

Comparator-stratified analyses suggested that glycemic effects varied by control condition, but these findings should be interpreted cautiously because the comparator categories addressed different clinical questions and were not directly interchangeable. In placebo-controlled trials, significant reductions were observed for both FPG (MD −17.59 mg/dL, 95% CI −30.52 to −4.66; *I*^2^ = 98.92%) and PPG (MD −23.15 mg/dL, 95% CI −38.46 to −7.83; *I*^2^ = 93.12%). Trials using metformin as the comparator did not show clear improvements in FPG (MD −6.71 mg/dL, 95% CI −21.01 to 7.59; *I*^2^ = 93.77%) and yielded a directionally unfavorable estimate for PPG (MD 11.44 mg/dL, 95% CI −16.89 to 39.77; *I*^2^ = 97.38%), reflecting the different interpretation of comparisons against active pharmacological therapy. Trials categorized as “other comparators”, including food controls, active controls, or pragmatic control conditions, showed reductions in FPG (MD −12.87 mg/dL, 95% CI −24.99 to −0.75; *I*^2^ = 92.70%) and PPG (MD −29.53 mg/dL, 95% CI −36.32 to −22.75; *I*^2^ = 10.01%). The test for subgroup differences was not significant for FPG but was significant for PPG, indicating that comparator type may have contributed to variation in PPG effects (Figure 8). For HbA1c, a significant reduction was observed only in the subgroup with other comparators (MD −0.47%, 95% CI −0.85 to −0.10; *I*^2^ = 96.39%), whereas placebo- and metformin-controlled trials did not show significant effects. Because the “other comparator” category included clinically diverse control conditions, these subgroup findings should be interpreted cautiously (Supplementary Table S2).

#### 3.5.4. Treatment Duration and Publication Period

**Figure 9:**
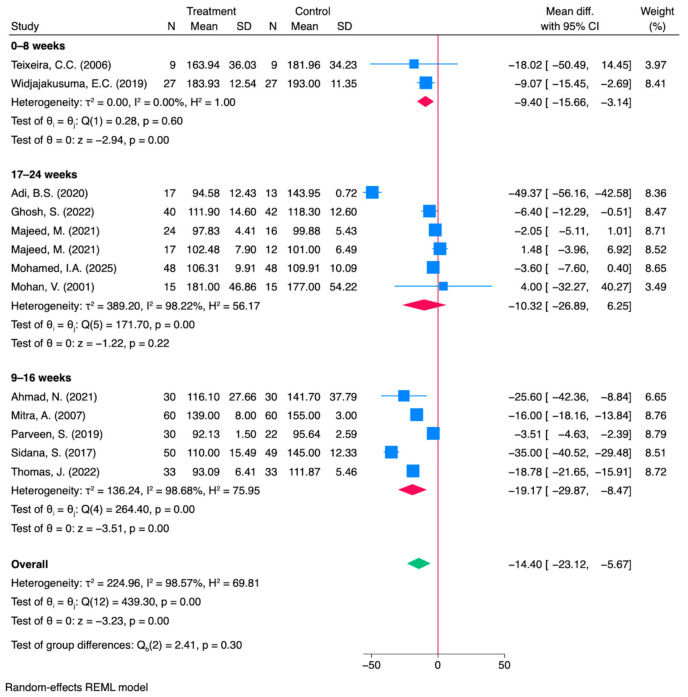
Subgroup forest plot analysis for FPG stratified by types of treatment duration. Random-effects meta-analyses were performed using REML. Pooled effects are expressed as MD with 95% CI [16,24,25,26,27,28,29,30,31,32,33,34]. Blue squares, individual study MDs, with square size proportional to study weight; horizontal lines, 95% CIs; red diamonds, subgroup pooled MDs; green diamond, pooled MD; red vertical line, line of no effect.

Duration-stratified analyses suggested that effects varied across outcomes, but between-subgroup differences by treatment duration were not statistically significant for FPG, PPG, or HbA1c. For FPG, significant reductions were observed in studies lasting 0–8 weeks (MD −9.40 mg/dL, 95% CI −15.66 to −3.14; *I*^2^ = 0.00%) and 9–16 weeks (MD −19.17 mg/dL, 95% CI −29.87 to −8.47; *I*^2^ = 98.68%). In contrast, studies lasting 17–24 weeks showed a non-significant reduction in FPG (Figure 9). For PPG, significant reduction was observed only in the 0–8-week subgroup, which also showed the lowest heterogeneity (MD −22.18 mg/dL, 95% CI −31.01 to −13.40; *I*^2^ = 5.18%). For HbA1c, a significant reduction was observed only in the 9–16-week subgroup (MD −0.44%, 95% CI −0.85 to −0.04; *I*^2^ = 95.76%), whereas other duration strata did not show significant changes (Supplementary Table S2).

When stratified by publication period, FPG reductions were observed across all publication periods. Significant reductions were observed in studies published during 2001–2010 (MD −15.94 mg/dL, 95% CI −18.09 to −13.79; *I*^2^ = 0.00%), 2011–2020 (MD −24.12 mg/dL, 95% CI −45.34 to −2.90; *I*^2^ = 98.87%), and 2021–2025 (MD −7.87 mg/dL, 95% CI −15.22 to −0.52; *I*^2^ = 93.72%). For PPG, a significant reduction was seen in 2011–2020 (MD −24.86 mg/dL, 95% CI −40.81 to −8.92; *I*^2^ = 96.00%), whereas the estimates for 2001–2010 and 2021–2025 were not significant. For HbA1c, a significant reduction was observed only in 2001–2010 (MD −0.20%, 95% CI −0.29 to −0.11; *I*^2^ = 0.00%), with no clear effect in later publication periods (Supplementary Table S2).

#### 3.5.5. Meta-Regression Analysis

**Figure 10:**
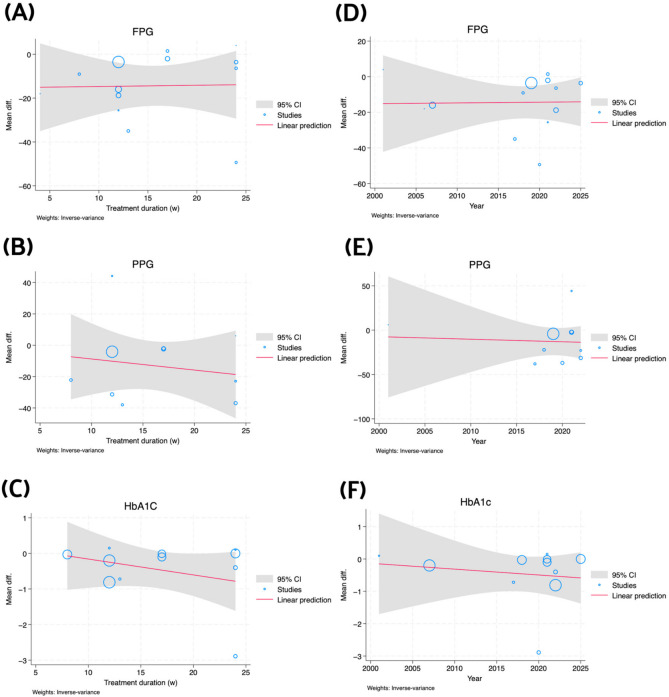
Meta-regression of glycemic outcomes by treatment duration (**A**–**C**) and publication year (**D**–**F**): FPG (**A**,**D**), PPG (**B**,**E**), and HbA1c (**C**,**F**).

Random-effects meta-regression (REML) was conducted to explore whether treatment duration and publication year explained between-study variability in glycemic effects. Treatment duration was not significantly associated with effect size for FPG (β = 0.057 mg/dL per week, 95% CI −1.457 to 1.570; *p* = 0.941), PPG (β = −0.707 mg/dL per week, 95% CI −3.560 to 2.146; *p* = 0.627), or HbA1c (β = −0.045% per week, 95% CI −0.135 to 0.046; *p* = 0.334). Similarly, publication year was not significantly associated with effect size for FPG (β = 0.042 mg/dL per year, 95% CI −1.450 to 1.534; *p* = 0.956), PPG (β = −0.289 mg/dL per year, 95% CI −3.924 to 3.346; *p* = 0.876), or HbA1c (β = −0.018% per year, 95% CI −0.103 to 0.067; *p* = 0.675) (Figure 10).

### 3.6. Effects on Lipid Profile

#### 3.6.1. Total Cholesterol, Triglycerides, and HDL

**Figure 11:**
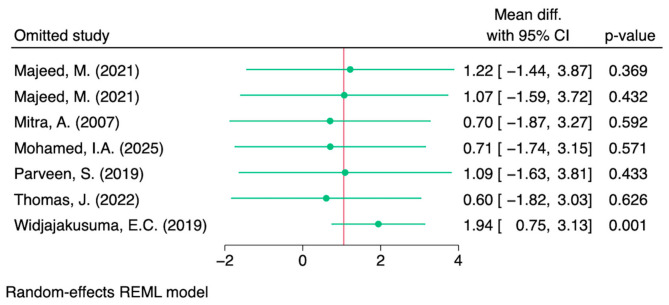
Leave-one-out sensitivity analysis for HDL. Influence analysis showing the pooled MD after omitting each study sequentially [27,28,29,31,33,34]. Green circles and horizontal lines, omission-specific pooled MDs and 95% CIs; red vertical line, overall pooled MD from all included studies.

Seven RCTs, including 239 participants in the intervention groups and 217 participants in the control groups, contributed lipid outcomes. Pooled analyses showed that Myrtaceae interventions were not associated with statistically significant changes in total cholesterol (MD −7.69 mg/dL, 95% CI −19.60 to 4.22; *p* = 0.206; *I*^2^ = 97.84%), triglycerides (MD −4.42 mg/dL, 95% CI −17.90 to 9.07; *p* = 0.521; *I*^2^ = 96.55%), or HDL (MD 1.05 mg/dL, 95% CI −1.21 to 3.32; *p* = 0.362; *I*^2^ = 94.04%) (Supplementary Figures S4–S6). The very high heterogeneity indicates substantial between-study variability and limits confidence in the pooled lipid estimates. Leave-one-out analyses did not materially alter conclusions for total cholesterol and triglycerides, with pooled estimates remaining non-significant after sequential omission of each study. For HDL-cholesterol, the overall estimate remained stable; however, omitting the influential study by Widjajakusuma, E.C. (2019) [34] shifted the pooled estimate toward a statistically significant increase (Figure 11).

#### 3.6.2. Subgroup Analyses of Lipid Outcomes

By participant type, total cholesterol was significantly reduced in participants with T2DM (MD −11.38 mg/dL, 95% CI −22.54 to −0.23; *I*^2^ = 95.26%), but not in participants with prediabetes (MD −5.16 mg/dL, 95% CI −25.32 to 15.00; *I*^2^ = 97.14%). HDL increased significantly in the prediabetes subgroup (MD 1.99 mg/dL, 95% CI 0.41 to 3.56; *I*^2^ = 55.45%), but not in T2DM. However, the between-subgroup differences by participant type were not statistically significant for total cholesterol, triglycerides, or HDL.

By plant species, other Myrtaceae interventions were associated with a significant reduction in total cholesterol (MD −17.15 mg/dL, 95% CI −33.48 to −0.82; *I*^2^ = 95.26%), whereas the *S. cumini* subgroup was not statistically significant. Conversely, a small increase in HDL was observed in the *S. cumini* subgroup (MD 1.48 mg/dL, 95% CI 0.22 to 2.73; *I*^2^ = 73.25%), but not in other Myrtaceae interventions. Analyses by plant part showed no statistically significant between-subgroup differences for total cholesterol, triglycerides, or HDL. By formulation composition, single-botanical interventions were associated with a modest increase in HDL-cholesterol (MD 2.46 mg/dL, 95% CI 0.24 to 4.68; *I*^2^ = 66.57%), whereas mixed formulations showed no clear effect on HDL.

**Figure 12:**
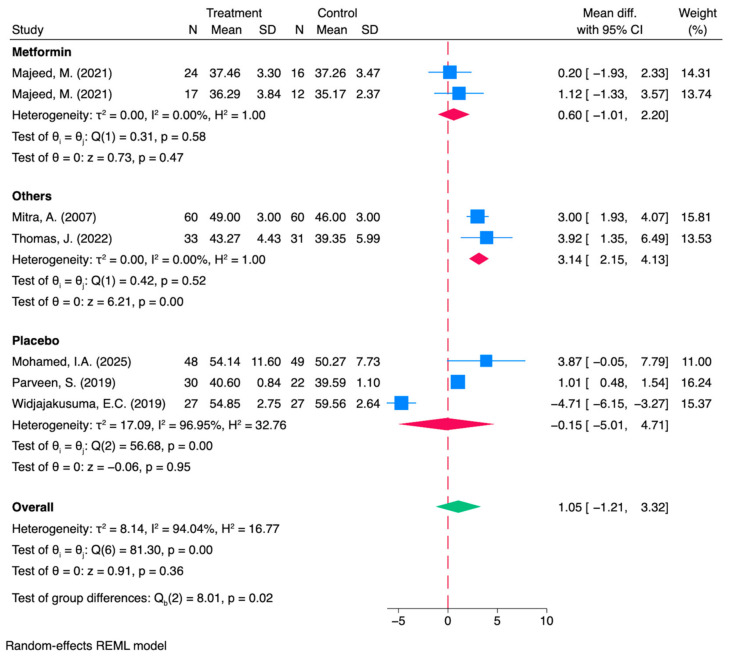
Subgroup forest plot analysis for HDL stratified by types of comparators. Random-effects meta-analyses were performed using REML. Pooled effects are expressed as MD with 95% CI [27,28,29,31,33,34]. Blue squares, individual study MDs, with square size proportional to study weight; horizontal lines, 95% CIs; red diamonds, subgroup pooled MDs; green diamond, pooled MD; red dashed vertical line, line of no effect.

Comparator-stratified analyses showed the clearest subgroup differences for lipid outcomes, although interpretation is limited because comparator categories were clinically diverse. In the other comparator subgroup, which included the soybean-based food control and the active control s-GSH, significant reductions were observed in total cholesterol (MD −25.54 mg/dL, 95% CI −38.88 to −12.19; *I*^2^ = 94.84%) and triglycerides (MD −27.87 mg/dL, 95% CI −29.98 to −25.76; *I*^2^ = 0.00%), together with an increase in HDL-cholesterol (MD 3.14 mg/dL, 95% CI 2.15 to 4.13; *I*^2^ = 0.00%). By treatment duration, HDL-cholesterol increased in the 9–16-week subgroup (MD 2.38 mg/dL, 95% CI 0.68 to 4.08; *I*^2^ = 84.64%), although the between-subgroup difference by duration was not significant. Overall, the lipid subgroup findings should be interpreted as exploratory because several strata included few studies and heterogeneity remained high (Figure 12 and Supplementary Table S3).

### 3.7. Safety Outcomes

#### 3.7.1. AST, ALT, and Creatinine

Seven RCTs, comprising 185 participants in the treatment groups and 163 participants in the control groups, reported AST and ALT. Six RCTs, consisting of 137 participants in the treatment groups and 114 participants in the control groups, reported serum creatinine levels. Pooled analyses indicated that Myrtaceae interventions were not associated with statistically significant changes in liver enzymes or renal function. Specifically, there was no between-group difference in AST (MD 0.60 U/L, 95% CI −1.08 to 2.29; *p* = 0.481; *I*^2^ = 42.40%), ALT (MD −1.51 U/L, 95% CI −3.26 to 0.24; *p* = 0.090; *I*^2^ = 60.33%), or serum creatinine (MD −0.02 mg/dL, 95% CI −0.14 to 0.10; *p* = 0.786; *I*^2^ = 92.57%). Leave-one-out analyses did not materially alter these safety conclusions (Supplementary Figures S7–S9). These findings indicate no clear short-term laboratory signal of hepatic or renal dysfunction in the available trials; however, they should not be interpreted as evidence of long-term safety because follow-up was short, sample sizes were small, and adverse-event reporting was limited.

#### 3.7.2. Subgroup Analyses of Safety Outcomes

Subgroup analyses of safety outcomes showed no consistent hepatic or renal safety signal. For AST, most subgroup estimates were generally small and non-significant; however, a small increase was observed in the *S. cumini* subgroup (MD 3.77 U/L, 95% CI 0.87 to 6.66; *I*^2^ = 0.00%) and in metformin-comparator trials (MD 4.09 U/L, 95% CI 1.05 to 7.14; *I*^2^ = 0.00%), with significant between-subgroup differences by species and comparator type. For ALT, small reductions were observed in the prediabetes subgroup (MD −2.77 U/L, 95% CI −4.69 to −0.86; *I*^2^ = 36.11%) and in the other-comparator subgroup (MD −3.57 U/L, 95% CI −6.96 to −0.17; *I*^2^ = 12.91%), with significant between-subgroup differences. Creatinine estimates were generally close to null across subgroups, although heterogeneity remained high in several strata (Supplementary Table S4). Overall, these subgroup findings should be interpreted cautiously because safety data were sparse, follow-up was short, and several subgroups included few studies.

### 3.8. Publication Bias

**Figure 13:**
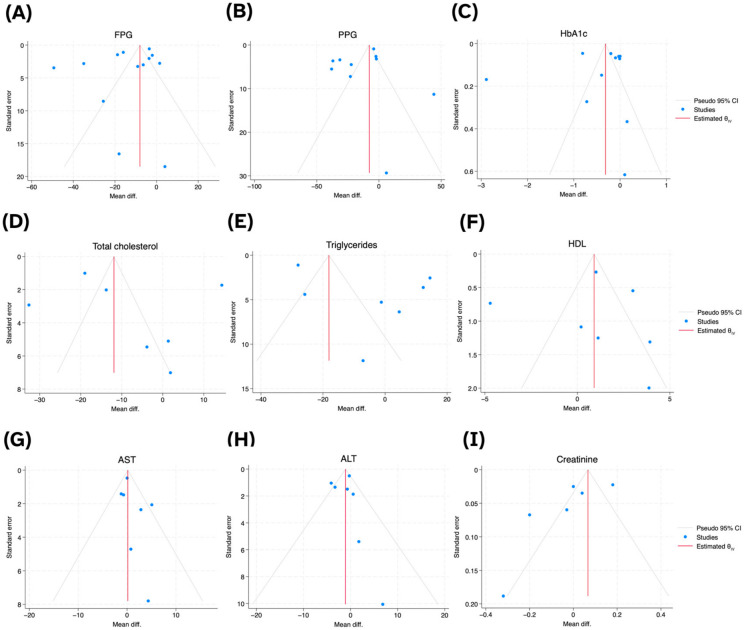
Funnel plot analysis for publication bias. Funnel plots for (**A**) FPG, (**B**) PPG, (**C**) HbA1c, (**D**) Total cholesterol, (**E**) Triglycerides, (**F**) HDL, (**G**) AST, (**H**) ALT, and (**I**) Creatinine. Effect size (MDs) are plotted against standard error.

Potential publication bias was assessed using visual inspection of funnel plot symmetry and a test for small-study effects. For the primary glycemic outcomes, there was no evidence of small-study effects for FPG, PPG, or HbA1c, and funnel plots were visually consistent with approximate symmetry. Similarly, no evidence of small-study effects was detected for lipid outcomes, including total cholesterol, triglycerides, and HDL-cholesterol. For laboratory safety outcomes, Egger’s test was also non-significant for AST and ALT. In contrast, for creatinine, Egger’s test suggested potential funnel plot asymmetry (*p* = 0.011) (Figure 13).

### 3.9. Certainty of Evidence

The certainty of evidence was assessed using the GRADE approach. Certainty was rated as low for FPG, AST, and ALT and very low for PPG, HbA1c, lipid outcomes, and creatinine. For FPG, the evidence was downgraded for risk of bias and inconsistency, but not for imprecision because the pooled estimate was statistically significant, the 95% CI did not cross the line of no effect, and leave-one-out sensitivity analysis showed a stable direction of effect. PPG, HbA1c, and lipid outcomes were rated as very low certainty because of risk-of-bias concerns, substantial heterogeneity, and imprecision. For laboratory safety markers, certainty was low for AST and ALT but very low for creatinine because of high heterogeneity, imprecision, and suspected small-study effects (Table 3).

## 4. Discussion

This is the first systematic review and meta-analysis of randomized controlled trials to comprehensively evaluate the efficacy and safety of *S. cumini* and related Myrtaceae interventions in prediabetes and T2DM. A key strength of this review is that it evaluated not only glycemic endpoints but also lipid parameters and laboratory safety markers, while further exploring potential sources of between-study variability through subgroup, sensitivity, meta-regression, and GRADE analyses. Across 13 randomized trials involving 802 participants, Myrtaceae interventions were associated with a significant reduction in FPG, whereas the pooled effects on PPG and HbA1c were not statistically significant. Effects on total cholesterol, triglycerides, and HDL-cholesterol were not significant overall, although selected subgroup analyses suggested possible modest benefits in some settings. Similarly, pooled analyses of AST, ALT, and creatinine showed no clear short-term laboratory signal for hepatic or renal dysfunction.

The significant reduction in FPG suggests a possible short-term glycemic signal for Myrtaceae interventions. In the present review, Myrtaceae interventions reduced FPG by 14.40 mg/dL compared with controls. However, this benefit should be interpreted cautiously because the certainty of evidence was low, the effect size was modest, and between-study heterogeneity was very high. Although leave-one-out analysis suggested that the finding was not driven by any single trial, the substantial heterogeneity limits confidence in the generalizability of the pooled estimate across species, formulations, comparator types, and participant subgroups. By comparison, a meta-analysis of chronic interventions with pure or enriched mixtures of (poly)phenols in individuals with T2DM or at risk of diabetes reported a more modest overall reduction in fasting glucose of 3.32 mg/dL, with only a slight reduction in HbA1c [36]. Similarly, Fallah et al. found that dietary anthocyanins significantly reduced fasting blood sugar by 2.70 mg/dL and 2 h PPG by 11.1 mg/dL, with stronger effects in diabetic participants and in trials lasting more than 8 weeks or using doses above 300 mg/day [37]. Yang et al. likewise reported that anthocyanin supplementation significantly improved fasting and 2 h PPG in randomized trials, supporting the view that polyphenol-rich botanical interventions can meaningfully influence short-term glycemic indices [38]. However, Rambaran et al. found no clear overall effect of berry polyphenols on biomarkers of glucose metabolism, highlighting that results may vary depending on botanical source, phytochemical composition, dose, study duration, and baseline metabolic status [39].

A major interpretive point is the difference between the FPG signal and the weaker evidence for PPG and HbA1c. In the present analysis, PPG showed a directionally favorable but statistically non-significant pooled effect, and the leave-one-out analysis indicated that statistical significance was sensitive to study composition. Therefore, the PPG finding should not be interpreted as a robust class-level effect. HbA1c was also not significantly improved in the overall analysis. This pattern is biologically plausible and methodologically unsurprising, because HbA1c reflects average glycemia over approximately the preceding 8 to 12 weeks and is therefore less responsive than FPG or PPG to brief interventions, modest metabolic changes, and clinically heterogeneous populations [40]. In the present review, many included trials were short in duration, which would be expected to favor detection of short-term changes in circulating glucose rather than durable changes in integrated glycemic burden. This interpretation is partly consistent with previous studies on polyphenol and anthocyanin synthesis. Palma-Duran et al. found that polyphenol supplementation significantly lowered HbA1c overall and in participants with T2DM, but not in non-diabetic or prediabetic populations, suggesting that metabolic phenotype and baseline glycemic severity may strongly influence the likelihood of detecting HbA1c improvement [41]. Likewise, Fallah et al. reported significant reductions in FPG, 2 h PPG, and HbA1c with dietary anthocyanins, particularly when interventions lasted longer than 8 weeks, used doses above 300 mg/day, and were administered to patients with T2DM [37]. More recently, Mao et al. showed that anthocyanin supplementation in T2DM significantly reduced HbA1c, FPG, and 2 h PPG, supporting the view that more standardized, anthocyanin-focused interventions in clearly diabetic populations may be more likely to influence both short- and intermediate-term glycemic markers [42]. In contrast, Macena et al. reported only a modest HbA1c reduction in patients with diabetic nephropathy, while emphasizing that the overall evidence was limited by a high risk of bias and low or very low certainty [43]. Against this background, our findings suggest that Myrtaceae interventions may retain a short-term FPG-lowering signal, but evidence for PPG and HbA1c remains uncertain.

Mechanistically, *S. cumini* and related Syzygium species consistently identify a phytochemical profile rich in anthocyanins, ellagic and gallic acid derivatives, flavonoids, tannins, and other phenolic compounds with plausible antihyperglycemic activity [8,10,44]. More generally, dietary polyphenols can attenuate postprandial hyperglycemia by inhibiting α-amylase and α-glucosidase, delaying starch hydrolysis, reducing intestinal glucose uptake, and altering incretin- and transporter-related handling of carbohydrate [12,13,14]. This may be particularly relevant to trials evaluating guava, jaboticaba, and clove-based interventions, where effects on intestinal glucose handling, postprandial metabolism, or hepatic glucose regulation have been proposed [44,45,46,47]. Beyond carbohydrate digestion and absorption, Myrtaceae phytochemicals may influence pathophysiologic processes relevant to dysglycemia. Experimental and translational studies of *S. cumini* indicate effects on insulin sensitivity, β-cell function, glucose transport, oxidative stress, and inflammatory signaling [17,48]. Preclinical studies have reported that *S. cumini* leaf or seed preparations can improve insulin resistance, reduce TNF-α and oxidative stress, influence PPARγ-related pathways, enhance glucose transporter expression, and support pancreatic islet function [49,50]. Other work has shown antiglycation, antioxidant, and digestive enzyme-inhibitory properties of *S. cumini* fractions [51]. These observations align with the broader diabetes literature, in which oxidative stress, chronic low-grade inflammation, AGE-RAGE signaling, and β-cell dysfunction are now recognized as major drivers of insulin resistance, progressive dysglycemia, and diabetic complications [15,52,53]. These mechanisms are biologically plausible, but they should not be interpreted as direct evidence of clinical efficacy. Effects observed in experimental or translational studies may not translate consistently to humans because of differences in dose, bioavailability, formulation, metabolic phenotype, and treatment duration.

In our subgroup analyses, several findings appeared biologically plausible, including stronger FPG reductions in T2DM than in prediabetes, variation by plant part, and larger point estimates in some single-botanical strata. However, these subgroup findings should be interpreted as exploratory and hypothesis-generating rather than confirmatory. Many subgroup strata included few studies, and residual heterogeneity remained substantial. Evidence from single-botanical trials provides a more direct estimate of the effect of the tested Myrtaceae species, whereas evidence from mixed or polyherbal formulations is less direct because other ingredients may have independent or synergistic metabolic effects. When *S. cumini* or another Myrtaceae species is co-formulated with other plants that themselves have glucose-lowering potential, any apparent benefit cannot be attributed confidently to the Myrtaceae component alone. This problem is common in ethnomedicine research and has been repeatedly highlighted in broader reviews of plant-based interventions for diabetes [54,55,56]. Differences by plant part are also plausible, as the phytochemical composition and putative antidiabetic activity of *S. cumini* and related species may vary across seeds, leaves, fruits, and other plant parts [8,10]. These findings may help inform future trial design but should not be over-interpreted as definitive evidence of effect modification.

Comparator type also materially affects interpretation. Placebo-controlled trials, metformin-comparator trials, food controls, active controls, and pragmatic control conditions address different clinical questions and are not directly interchangeable. It is unsurprising that effects versus placebo or pragmatic controls may appear larger than effects versus established pharmacological therapy [57]. In particular, the “other comparator” category combined clinically diverse control conditions; therefore, favorable findings in this subgroup should be considered exploratory and should not be interpreted as evidence of superiority over standard pharmacological therapy. Accordingly, the present findings are more consistent with a possible adjunctive role than a replacement role for Myrtaceae interventions. The subgroup analyses also help explain why heterogeneity remained high in the overall models. Across the included trials, studies differed not only in species and plant part but also in formulation, dose, duration, comparator intensity, baseline phenotype, and concomitant background therapy. Methodological guidance for systematic reviews emphasizes that this clinical heterogeneity should be explicitly investigated, because pooled estimates may obscure clinically meaningful variation in who benefits, from which intervention, and under what conditions [58,59]. At the same time, subgroup analyses are inherently vulnerable to both false-positive and false-negative findings, especially when multiple comparisons are performed or when few studies contribute to each subgroup [60,61]. In the present review, subgroup findings are clinically plausible and useful for interpretation as hypothesis-generating rather than confirmatory evidence of effect modifiers, unless they are supported by a strong biological rationale, consistency across related outcomes, and adequate between-study information.

Although the categorical subgroup analyses suggested that treatment duration and publication period might influence the magnitude of effect, the continuous meta-regression models did not identify significant associations for either treatment duration or publication year. This apparent inconsistency is not unusual in evidence synthesis. Study-level meta-regression is often underpowered when only a small number of studies contribute to an outcome, and in such settings the regression may fail to detect threshold effects, non-linear associations, or context-dependent differences that are more visible in clinically defined strata [62,63]. In addition, meta-regression based on aggregate study characteristics is susceptible to ecological fallacy, overfitting, and other methodological pitfalls, meaning that relationships observed across studies may not reflect patient-level or intervention-level causal effects [64]. Accordingly, the null meta-regression results do not negate the subgroup findings; rather, they indicate that the currently available evidence is insufficient to support strong claims about linear duration–response or time-trend effects, and that any apparent modifiers should be interpreted as provisional signals requiring confirmation in larger, more standardized trials.

The lipid findings were less convincing than the glycemic findings. Although selected subgroup analyses suggested modest increases in HDL-cholesterol or reductions in lipid parameters in selected strata, the overall pooled estimates for total cholesterol, triglycerides, and HDL-cholesterol were neutral and heterogeneity was very high. This contrasts with several broader syntheses of anthocyanin- and polyphenol-rich interventions, in which at least some lipid fractions improved significantly. For example, recent meta-analyses of anthocyanin supplementation have reported significant improvements in triglycerides, LDL-cholesterol, and HDL-cholesterol [65,66]. The weaker and less consistent lipid signal in our review likely reflects the marked diversity of the included interventions, comparators, and treatment durations. Mechanistically, this is plausible because polyphenol-rich botanicals do not influence lipid metabolism through a single pathway. Instead, their effects may depend on the phytochemical composition and dose, with proposed mechanisms including suppression of hepatic lipogenesis, activation of AMPK- and PPAR-related pathways, enhancement of fatty acid oxidation, inhibition of intestinal cholesterol absorption, and promotion of reverse cholesterol transport and cholesterol efflux [67,68]. These pathways are structurally and dose-dependent, which may explain why lipid effects are often less reproducible than glucose-related effects when chemically diverse preparations are pooled together.

In our review, pooled AST, ALT, and creatinine did not differ significantly between Myrtaceae and control groups. However, the number of reporting trials was small, treatment duration was generally brief, and safety was often a secondary outcome rather than a rigorously prespecified endpoint. Accordingly, the findings indicate no clear short-term laboratory signal for hepatic or renal dysfunction within the available trials, but they should not be interpreted as evidence of long-term safety. The preclinical data suggested that *S. cumini* may exert hepatoprotective and renoprotective effects by attenuating oxidative stress, improving antioxidant defenses, and activating Nrf2-dependent cytoprotective signaling [69,70]. However, the broader literature on herbal and dietary supplements indicated that botanical products could cause idiosyncratic liver injury, ranging from transient liver enzyme elevations to severe hepatotoxicity [71,72,73]. This is especially important in a body of literature where product composition, extraction methods, co-ingredients, and quality control may vary substantially across studies and commercial formulations. Future trials should therefore include longer follow-up, systematic adverse-event monitoring, and prespecified hepatic and renal safety outcomes.

A major challenge in this review was the degree of heterogeneity. Heterogeneity was extreme for the main glycemic and lipid outcomes and often remained substantial even after subgrouping. Clinically, this was expected. The included studies varied in species, plant part, formulation, extraction method, dose, treatment duration, background therapy, comparator intensity, and participant phenotype. Methodological variability added further complexity, including small sample sizes, inconsistent reporting of outcomes, differing blinding practices, and limited protocol transparency. We attempted to address these issues using random-effects modeling, prespecified subgroup analyses, meta-regression, and leave-one-out sensitivity analyses. Even so, residual heterogeneity remained high, indicating that the pooled estimates should be interpreted as average effects across heterogeneous Myrtaceae-based interventions rather than a single uniform treatment effect. This is a familiar pattern in botanical and ethnomedicine research, where promising signals often coexist with substantial design and reporting limitations [54,55,74]. The GRADE assessment further supports cautious interpretation. The certainty of evidence was low for FPG, AST, and ALT and very low for PPG, HbA1c, lipid outcomes, and creatinine. Certainty was downgraded primarily because no included trial was judged as having overall low risk of bias, heterogeneity was substantial or very high, several estimates were imprecise, and safety reporting was limited. Therefore, although the FPG finding suggests a possible short-term glycemic signal, the current evidence is insufficient to support definitive conclusions regarding broader glycemic efficacy, lipid improvement, or long-term safety.

Several limitations should be prioritized when interpreting these findings. First, the certainty of the evidence was low to very low across outcomes, mainly because no included trial was judged to have an overall low risk of bias, several estimates were imprecise, and safety reporting was limited. Second, substantial clinical and statistical heterogeneity remained, reflecting differences in participant phenotype, Myrtaceae species, plant part, formulation, dose, comparator type, and treatment duration; therefore, pooled estimates should be interpreted as average effects across heterogeneous interventions rather than as uniform treatment effects [75,76]. Third, intervention standardization was limited, particularly for mixed or polyherbal formulations in which the Myrtaceae-specific contribution could not be isolated. In addition, homeopathic preparations were not analyzed as a separate intervention category; therefore, their contribution should be considered part of the broader intervention heterogeneity. Finally, safety conclusions are limited by short follow-up, sparse adverse-event reporting, and reliance on laboratory markers rather than systematic clinical safety assessment. These limitations restrict clinical generalizability and highlight the need for more rigorous, product-specific evidence.

From a clinical and translational perspective, the present findings do not support the use of Myrtaceae interventions as substitutes for established pharmacotherapy in dysglycemia. Rather, the most plausible application is as a carefully selected adjunct to standard care, particularly in adults with prediabetes or early T2DM who remain under routine clinical monitoring and in whom treatment intensification may be delayed or limited by therapeutic inertia or medication intolerance [3,5,6]. In practice, this means that only well-characterized and standardized products should be considered for further clinical translation, because complete reporting of herbal interventions and consistent quality control are essential for meaningful interpretation and reproducibility [58,59]. At the same time, the generalizability of our findings is limited because most included trials were small, short, and conducted in a limited number of countries with substantial variation in botanical composition, preparation, and comparator intensity. Accordingly, clinicians and researchers should not extrapolate these findings to all commercial jamun, clove, or other Myrtaceae products, because herbal and botanical products can vary substantially in composition, manufacturing quality, and reporting of critical intervention characteristics. Translation into practice should therefore remain cautious, product-specific, and ideally limited to settings in which formulation quality, dosing, and follow-up can be adequately controlled [77,78].

## 5. Conclusions

This systematic review and meta-analysis suggests that *Syzygium cumini* and related Myrtaceae interventions may provide a modest short-term reduction in FPG among adults with T2DM or prediabetes. However, evidence for PPG was directionally favorable but statistically unstable, and current evidence does not support a consistent benefit for HbA1c or lipid outcomes. Pooled laboratory safety markers showed no clear short-term signal for hepatic or renal dysfunction, but the available evidence is insufficient to establish long-term safety. The findings should be interpreted cautiously because effects appear likely to be preparation-specific rather than a uniform Myrtaceae class effect, and evidence from mixed or polyherbal formulations cannot be attributed solely to the Myrtaceae component. Given the substantial heterogeneity, short follow-up, small sample sizes, absence of low-risk trials, and low to very-low certainty of evidence, these interventions should be regarded as promising but unproven adjuncts rather than alternatives to standard dysglycemia management. Future trials should be adequately powered, longer in duration, species-specific, and focused on phytochemically standardized single-botanical preparations, with prespecified glycemic, lipid, and safety outcomes, systematic adverse-event monitoring, and clinically appropriate comparator groups.

## Figures and Tables

**Table 1:** Summary of search strategies.

Database	Search Strategy	Search Result
PubMed	(“Syzygium cumini”[Title/Abstract] OR “Eugenia jambolana”[Title/Abstract] OR “Syzygium jambolanum”[Title/Abstract] OR “jamun”[Title/Abstract] OR “jambul”[Title/Abstract] OR “jambolan”[Title/Abstract] OR “Java plum”[Title/Abstract] OR “Indian blackberry”[Title/Abstract] OR “Syzygium”[Title/Abstract] OR “Psidium guajava”[Title/Abstract] OR “Eugenia uniflora”[Title/Abstract] OR “Myrtus communis”[Title/Abstract] OR “Pimenta dioica”[Title/Abstract] OR “Eucalyptus globulus”[Title/Abstract] OR “Myrtaceae”[Title/Abstract] OR “Syzygium”[MeSH Terms] OR “Myrtaceae”[MeSH Terms]) AND (“diabetes mellitus”[MeSH Terms] OR “diabetes mellitus, type 2”[MeSH Terms] OR “diabetes mellitus, type 1”[MeSH Terms] OR “metabolic syndrome”[MeSH Terms] OR “hyperglycemia”[MeSH Terms] OR “hyperglyc*”[Title/Abstract] OR “insulin resist*”[Title/Abstract] OR “T2DM”[Title/Abstract] OR “T1DM”[Title/Abstract] OR “diabetes”[Title/Abstract])	520
Scopus	(TITLE-ABS-KEY (prediabet*)) OR (TITLE-ABS-KEY (“impaired glucose”)) OR (TITLE-ABS-KEY (“metabolic syndrome”)) OR (TITLE-ABS-KEY (hyperglyc*)) OR (TITLE-ABS-KEY (“insulin resist*”)) OR (TITLE-ABS-KEY (t2dm)) OR (TITLE-ABS-KEY (“type 2 diabetes”)) OR (TITLE-ABS-KEY (“type 1 diabetes”)) OR (TITLE-ABS-KEY (t1dm))) AND ((TITLE-ABS-KEY (“Syzygium cumini”)) OR (TITLE-ABS-KEY (“Eugenia jambolana”)) OR (TITLE-ABS-KEY (“Syzygium jambolanum”)) OR (TITLE-ABS-KEY (jamun)) OR (TITLE-ABS-KEY (jambul)) OR (TITLE-ABS-KEY (jambolan)) OR (TITLE-ABS-KEY (“Java plum”)) OR (TITLE-ABS-KEY (“indian blackberry”)) OR (TITLE-ABS-KEY (duhat)) OR (TITLE-ABS-KEY (Syzygium)) OR (TITLE-ABS-KEY (“Psidium guajava”)) OR (TITLE-ABS-KEY (“Eugenia uniflora”)) OR (TITLE-ABS-KEY (“Myrtus communis”)) OR (TITLE-ABS-KEY (“Pimenta dioica”)) OR (TITLE-ABS-KEY (“Eucalyptus globulus”)) OR (TITLE-ABS-KEY (Myrtaceae)))	567
Embase	(exp Syzygium cumini/OR exp Syzygium cumini extract/OR “Syzygium cumini”.mp. OR “Eugenia jambolana”.mp. OR “Syzygium jambolanum”.mp. OR Syzygium.mp. OR exp Syzygium/OR exp Syzygium polyanthum/OR exp Myrtus communis extract/OR “Myrtus communis”.mp. OR Myrtaceae.mp. OR exp Myrtaceae/) AND (exp non insulin dependent diabetes mellitus/OR “type 2 diabetes mellitus”.mp. OR exp insulin dependent diabetes mellitus/OR “type 1 diabetes mellitus”.mp. OR hyperglycemia.mp. OR exp hyperglycemia/OR exp postprandial hyperglycemia/OR exp impaired glucose tolerance/OR prediabet*.mp. OR diabetes.mp. OR exp diabetes mellitus/) AND (HbA1c.mp. OR exp hemoglobin A1c/OR exp glucose blood level/OR fasting plasma glucose.mp. OR exp glucose/OR exp insulin blood level/OR exp insulin/OR “fasting insulin”.mp. OR “lipid profile”.mp. OR “adverse events”.mp. OR exp adverse event/OR “blood pressure”.mp. OR exp blood pressure/)	682
MEDLINE	(“Syzygium cumini”.mp. OR exp Syzygium/OR “Eugenia jambolana”.mp. OR “Syzygium jambolanum”.mp. OR Myrtaceae.mp. OR exp Myrtaceae/) AND (“diabetes mellitus”.mp. OR exp diabetes mellitus/OR exp diabetes mellitus, type 2/OR T2DM.mp. OR exp diabetes mellitus, type 1/OR T1DM.mp. OR “metabolic syndrome”.mp. OR exp metabolic syndrome/OR hyperglycemia.mp. OR exp hyperglycemia/OR exp prediabetic state/OR prediabet*.mp.) AND (“fasting plasma glucose”.mp. OR exp glycated hemoglobin/OR exp blood glucose/OR HbA1c.mp. OR “fasting insulin”.mp. OR exp dyslipidemias/OR “lipid profile”.mp. OR exp cardiovascular diseases/OR exp cholesterol, HDL/OR exp cholesterol/OR exp cholesterol, LDL/OR exp cholesterol, VLDL/OR cholesterol.mp. OR exp triglycerides/OR triglyceride.mp.)	177
Web of Science	Topic = (“Syzygium cumini” OR “Eugenia jambolana” OR “Syzygium jambolanum” OR Syzygium OR “Psidium guajava” OR “Eugenia uniflora” OR “Myrtus communis” OR “Pimenta dioica” OR “Eucalyptus globulus” OR Myrtaceae) AND Topic = (diabetes OR prediabet* OR “impaired glucose” OR “metabolic syndrome” OR hyperglyc* OR “insulin resist*”) AND Topic = (HbA1c OR “fasting plasma glucose” OR “fasting blood glucose” OR “blood glucose level” OR “fasting insulin” OR “lipid profile” OR cholesterol OR triglyceride OR “adverse event”)	719

The asterisk (*) indicates a truncation symbol used to retrieve words with different endings.

**Table 2:** Study characteristics of included randomized controlled trials of *Syzygium cumini* and related Myrtaceae interventions.

Study	Country	Design	Population	Diagnosis/Eligibility	Intervention (Species; Part; Form; Composition)	Dose & Frequency	Duration	Comparator	N	Age (y)	M:F
Adi, B.S. (2020) [24]	India	Double-blind parallel RCT	T2DM	Blood sugar ≥ 7.0 mmol/L	*Syzygium cumini*; NR; homeopathic; single	30C potency, BID	24 wk	Placebo	30	30–60 (range)	12:18
Ahmad, N. (2021) [25]	India	Single-blind parallel RCT	T2DM	FPG 126–150 mg/dL and/or PPG 200–250 mg/dL and/or HbA1c ≥ 6.5%	*Syzygium cumini* (seed kernel); powder; mixed (Gymnema + Tinospora + Syzygium)	6 g BID (12 g/day)	12 wk	Metformin	60	47.7	36:24
Ghosh, S. (2022) [26]	India	Open-label parallel RCT	Prediabetes	IFG/FPG 100–125 mg/dL, IGT/OGTT 140–199 mg/dL, or HbA1c 5.7–6.4%	Homeopathic mother tinctures; individualized single-herb add-on (Cephalandra or Gymnema or Syzygium)	10–30 drops, 1–3×/day	24 wk	Active control (IHMs alone)	82	50.8 ± 8.1	37:45
Majeed, M. (2021) [27]	India	Double-blind parallel RCT	T2DM & Prediabetes	T2DM: HbA1c 6.5–7.5% + FBS > 125 mg/dL; PreDM: HbA1c 5.7–6.4% + FBS 100–125 mg/dL	GlycaCare-II^®^ (multi-ingredient incl. Syzygium fruit extract); tablets; mixed	522.5 mg/tablet, BID	17 wk	Metformin	69	50.45	33:36
Mitra, A. (2007) [28]	India	Single-blind parallel RCT	T2DM	FBS < 180 mg/dL	Multi-ingredient composite incl. jamun fruit + seed; aqueous extract/powder; mixed	Composite: 60 g fruit extract + 10 g seed, OD	12 wk	Food control (soybean-based)	120	48.29 ± 4.56	62:58
Mohamed, I.A. (2025) [29]	New Zealand	Double-blind parallel RCT	Prediabetes	BMI ≥ 26; FPG 5.6–6.9 mmol/L; Finnish risk score ≥ 12	*Acca sellowiana*; fruit; powder; single	1.15 g OD	24 wk	Placebo	97	46.4 ± 10.1	18:79
Mohan, V. (2001) [30]	India	Double-blind parallel RCT	T2DM	Secondary failure to oral hypoglycemic agents	Ayurvedic compound (incl. Syzygium + Gymnema + Cephalandra); tablets; mixed	2 tablets TID	24 wk	Placebo	30	55.00 ± 8.43	11:19
Parveen, S. (2019) [31]	India	Single-blind parallel RCT	Prediabetes	FBS 100–125 mg/dL, 2 h PG 140–199 mg/dL, or HbA1c 5.7–6.4%	*Syzygium cumini*; seed; capsules (powder); single	2.25 g/day (0.75 g × 3 caps), BID	12 wk	Placebo	52	40.33 ± 12.40	25:27
Sidana, S. (2017) [32]	India	Double-blind parallel RCT	T2DM	FBS > 126 mg/dL; PPBS > 180 mg/dL	*Syzygium cumini*; seed; powder; single	5 g BID (10 g/day)	12 wk	Placebo	99	NR	NR
Teixeira, C.C. (2006) [16]	Brazil	Double-blind parallel RCT (3-arm)	T2DM	FPG 7–11 mmol/L	*Syzygium cumini*; leaf; tea; single	2.0 g dry leaf/L tea, OD	4 wk	Placebo/ glyburide	27	56.5 ± 8.0	17:10
Thomas, J. (2022) [33]	India	Double-blind parallel RCT	Prediabetes	FBS 100–125 mg/dL or PPBS 140–199 mg/dL and HbA1c 5.7–6.4%	*Syzygium aromaticum*; clove bud; capsule; single	250 mg OD	12 wk	Active control (s-GSH)	70	44.00 ± 7.62	NR
Widjajakusuma, E.C. (2019) [34]	Indonesia	Double-blind parallel RCT	T2DM (on metformin)	T2DM with metformin treatment	*Syzygium polyanthum* + Andrographis; leaf; tablet; mixed	450 mg BID	8 wk	Placebo	54	53.15 ± 9.61	22:32
Widyawati, T. (2021) [35]	Indonesia	Parallel RCT	T2DM (on metformin)	T2DM with metformin treatment	*Syzygium polyanthum*; leaf; capsule; single	350 mg OD	2 wk	Placebo	12	NR	NR

Abbreviations: BID, twice daily; OD, once daily; TID, three times daily; FPG/FBS, fasting plasma/blood glucose; PPG/PPBS, postprandial glucose; OGTT, oral glucose tolerance test; NR, not reported.

**Table 3:** GRADE summary of findings.

Outcome	Studies, *n*	Participants, *n*	Effect Estimate, MD (95% CI)	*I*^2^ (%)	Risk of Bias	Inconsistency	Indirectness	Imprecision	Publication Bias	Certainty of Evidence
FPG	13	776	−14.40 mg/dL (−23.12 to −5.67)	98.57	Serious	Serious	Not serious	Not serious	Not serious	⨁⨁◯◯ Low
PPG	10	542	−12.99 mg/dL (−27.74 to 1.76)	97.93	Serious	Serious	Not serious	Serious	Not serious	⨁◯◯◯ Very low
HbA1c	11	707	−0.46% (−0.98 to 0.06)	99.20	Serious	Serious	Not serious	Serious	Not serious	⨁◯◯◯ Very low
Total cholesterol	7	456	−7.69 mg/dL (−19.60 to 4.22)	97.84	Serious	Serious	Not serious	Serious	Not serious	⨁◯◯◯ Very low
Triglycerides	7	456	−4.42 mg/dL (−17.90 to 9.07)	96.55	Serious	Serious	Not serious	Serious	Not serious	⨁◯◯◯ Very low
HDL	7	456	1.05 mg/dL (−1.21 to 3.32)	94.04	Serious	Serious	Not serious	Serious	Not serious	⨁◯◯◯ Very low
AST	7	348	0.60 U/L (−1.08 to 2.29)	42.40	Serious	Not serious	Not serious	Serious	Not serious	⨁⨁◯◯ Low
ALT	7	348	−1.51 U/L (−3.26 to 0.24)	60.33	Serious	Not serious	Not serious	Serious	Not serious	⨁⨁◯◯ Low
Creatinine	6	251	−0.02 mg/dL (−0.14 to 0.10)	92.57	Serious	Serious	Not serious	Serious	Serious	⨁◯◯◯ Very low

Abbreviations: ALT, alanine aminotransferase; AST, aspartate aminotransferase; CI, confidence interval; FPG, fasting plasma glucose; GRADE, Grading of Recommendations Assessment, Development and Evaluation; HbA1c, glycated hemoglobin; HDL, high-density lipoprotein cholesterol; MD, mean difference; PPG, postprandial glucose. GRADE certainty definitions: High certainty (⨁⨁⨁⨁), the true effect is likely to be close to the estimated effect; moderate certainty (⨁⨁⨁◯), the true effect is probably close to the estimated effect but may differ substantially; low certainty (⨁⨁◯◯), the true effect may differ substantially from the estimated effect; and very low certainty (⨁◯◯◯), the true effect is likely to differ substantially from the estimated effect.

## Data Availability

The original contributions presented in this study are included in the article/Supplementary Material. Further inquiries can be directed to the corresponding author.

## Supplementary Materials

The following supporting information can be downloaded at: https://www.mdpi.com/article/10.3390/foods15132332/s1, Figure S1: Leave-one-out sensitivity analysis for FPG. Influence analysis showing the pooled MD after omitting each study sequentially [16,24,25,26,27,28,29,30,31,32,33,34]. Green circles and horizontal lines, omission-specific pooled MDs and 95% CIs; red vertical line, overall pooled MD from all included studies. Figure S2: Leave-one-out sensitivity analysis for PPG. Influence analysis showing the pooled MD after omitting each study sequentially [24,25,26,27,30,31,32,33,34]. Green circles and horizontal lines, omission-specific pooled MDs and 95% CIs; red vertical line, overall pooled MD from all included studies. Figure S3: Leave-one-out sensitivity analysis for HbA1c. Influence analysis showing the pooled MD after omitting each study sequentially [24,25,26,27,28,29,30,32,33,34]. Green circles and horizontal lines, omission-specific pooled MDs and 95% CIs; red vertical line, overall pooled MD from all included studies. Figure S4: Forest plot of Myrtaceae interventions versus control for total cholesterol (mg/dL). Random-effects meta-analyses were performed using REML. Pooled effects are expressed as MD with 95% CI [27,28,29,31,33,34]. Blue squares, individual study MDs, with square size proportional to study weight; horizontal lines, 95% CIs; green diamond, pooled MD; red dashed vertical line, line of no effect. Figure S5: Forest plot of Myrtaceae interventions versus control for triglycerides (mg/dL). Random-effects meta-analyses were performed using REML. Pooled effects are expressed as MD with 95% CI [27,28,29,31,33,34]. Blue squares, individual study MDs, with square size proportional to study weight; horizontal lines, 95% CIs; green diamond, pooled MD; red dashed vertical line, line of no effect. Figure S6: Forest plot of Myrtaceae interventions versus control for HDL (mg/dL). Random-effects meta-analyses were performed using REML. Pooled effects are expressed as MD with 95% CI [27,28,29,31,33,34]. Blue squares, individual study MDs, with square size proportional to study weight; horizontal lines, 95% CIs; green diamond, pooled MD; red dashed vertical line, line of no effect. Figure S7: Leave-one-out sensitivity analysis for AST. Influence analysis showing the pooled MD after omitting each study sequentially [27,29,31,33,34,35]. Green circles and horizontal lines, omission-specific pooled MDs and 95% CIs; red vertical line, overall pooled MD from all included studies. Figure S8: Leave-one-out sensitivity analysis for ALT. Influence analysis showing the pooled MD after omitting each study sequentially [27,29,31,33,34,35]. Green circles and horizontal lines, omission-specific pooled MDs and 95% CIs; red vertical line, overall pooled MD from all included studies. Figure S9: Leave-one-out sensitivity analysis for creatinine. Influence analysis showing the pooled MD after omitting each study sequentially [27,31,33,34,35]. Green circles and horizontal lines, omission-specific pooled MDs and 95% CIs; red vertical line, overall pooled MD from all included studies. Table S1: Search strategies; Table S2: Subgroup analysis of glycemic outcomes; Table S3: Subgroup analysis of lipid outcomes; Table S4: Subgroup analysis of safety outcomes.

## Abbreviations

The following abbreviations are used in this manuscript:

T2DM	Type 2 diabetes mellitus
PRISMA	Preferred Reporting Items for Systematic Reviews and Meta-Analyses
PROSPERO	International Prospective Register of Systematic Reviews
MD	Mean difference
CI	Confidence interval
HbA1c	Glycated hemoglobin
IFG	Impaired fasting glucose
IGT	Impaired glucose tolerance
FPG	fasting plasma glucose
PPG	postprandial glucose
REML	restricted maximum likelihood
RoB 2	Cochrane Risk of Bias 2
BID	Twice daily
OD	Once daily
TID	Three times daily
FBS	Fasting blood sugar
PPBS	Postprandial blood glucose
OGTT	Oral glucose tolerance test
MetS	Metabolic syndrome
IHMs	Individualized homeopathic medicines
