# Supplementary material for: Efficacy and Safety of Syzygium cumini and Related Myrtaceae Interventions for Dysglycemia: A Systematic Review and Meta-Analysis of Randomized Controlled Trials
Source: Foods. 2026 Jul 1;15(13):2332. doi: 10.3390/foods15132332 (PMC13360929; doi:10.3390/foods15132332)
Supplement: Supplementary file 1 [file foods-15-02332-s001.zip › Supplementary Figures S1-S9.pdf]

Supplementary Figures

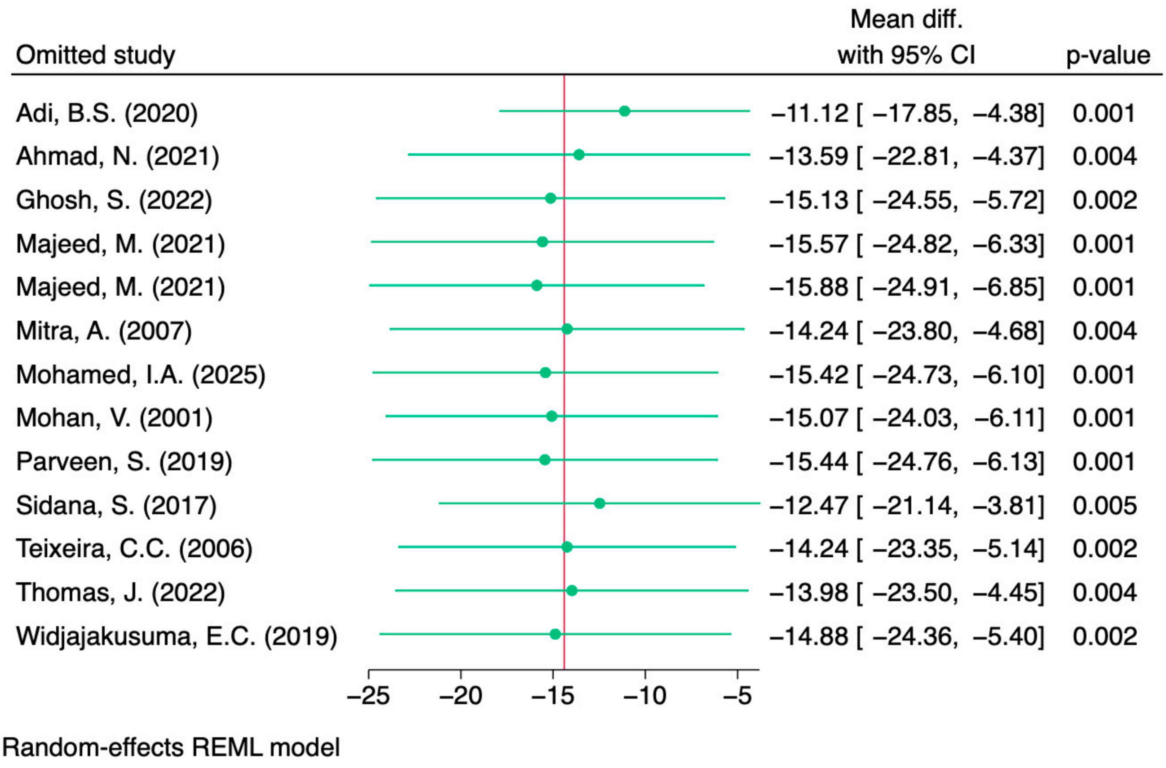

Figure S1. Leave-one-out sensitivity analysis for FPG. Influence analysis showing the pooled MD after omitting each study sequentially [16,24-34]. Green circles and horizontal lines, omission-specific pooled MDs and 95% CIs; red vertical line, overall pooled MD from all included studies.

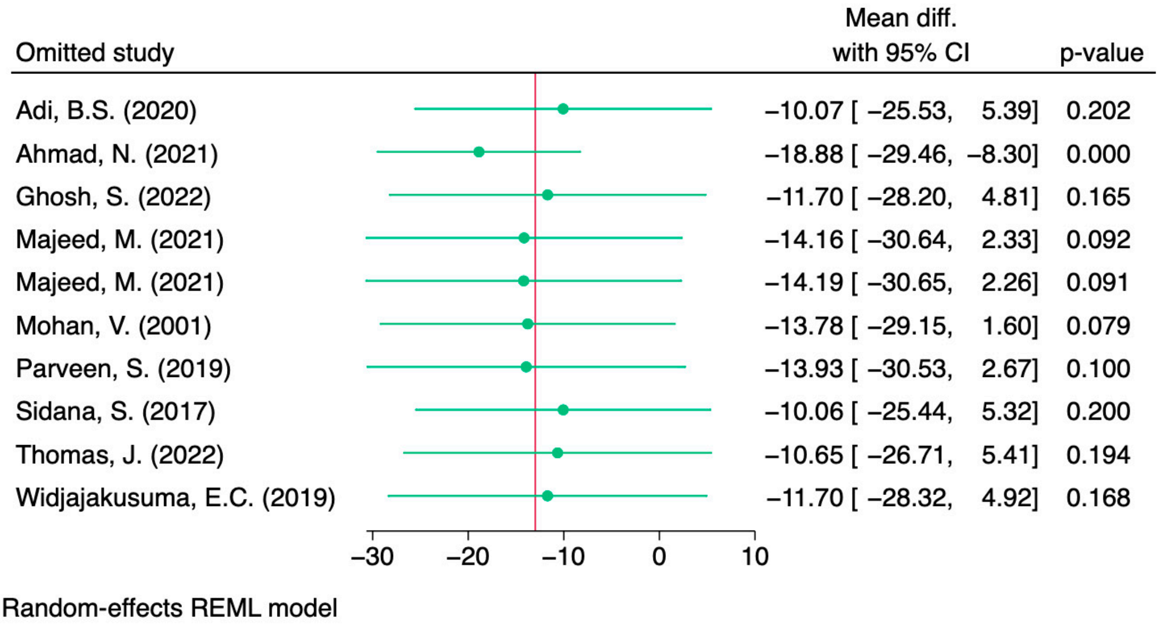

Figure S2. Leave-one-out sensitivity analysis for PPG. Influence analysis showing the pooled MD after omitting each study sequentially [24-27,30-34]. Green circles and horizontal lines, omission-specific pooled MDs and 95% CIs; red vertical line, overall pooled MD from all included studies.

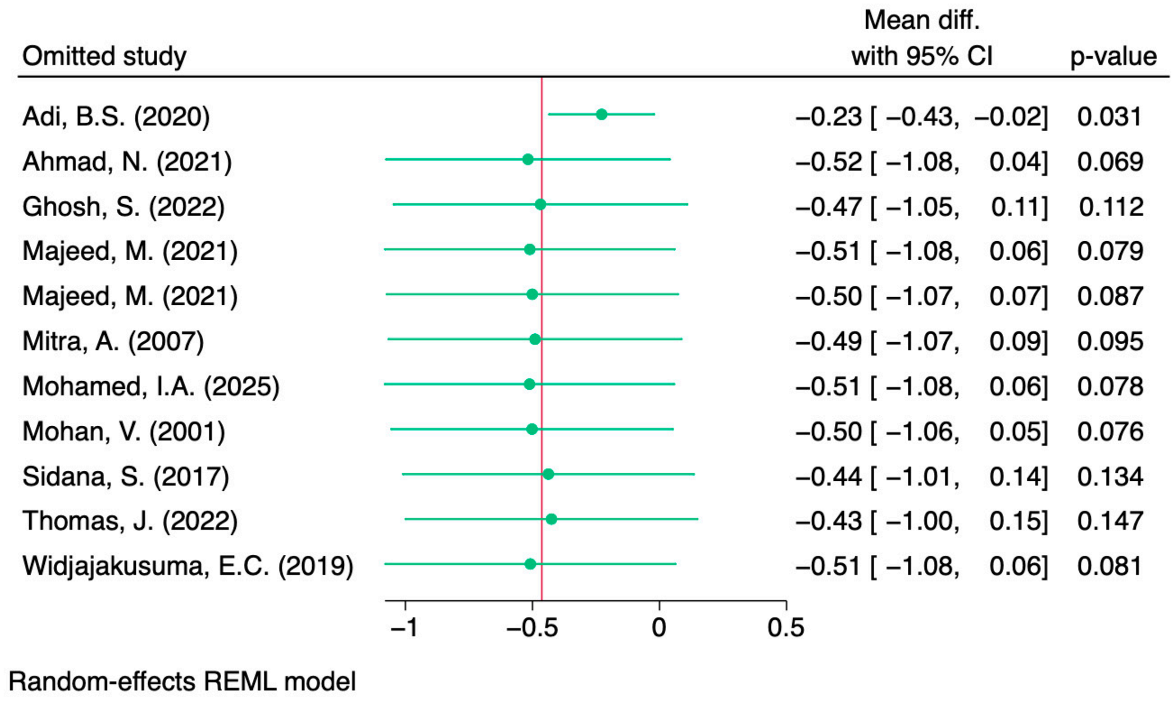

Figure S3. Leave-one-out sensitivity analysis for HbA1c. Influence analysis showing the pooled MD after omitting each study sequentially [24-30,32-34]. Green circles and horizontal lines, omission-specific pooled MDs and 95% CIs; red vertical line, overall pooled MD from all included studies.

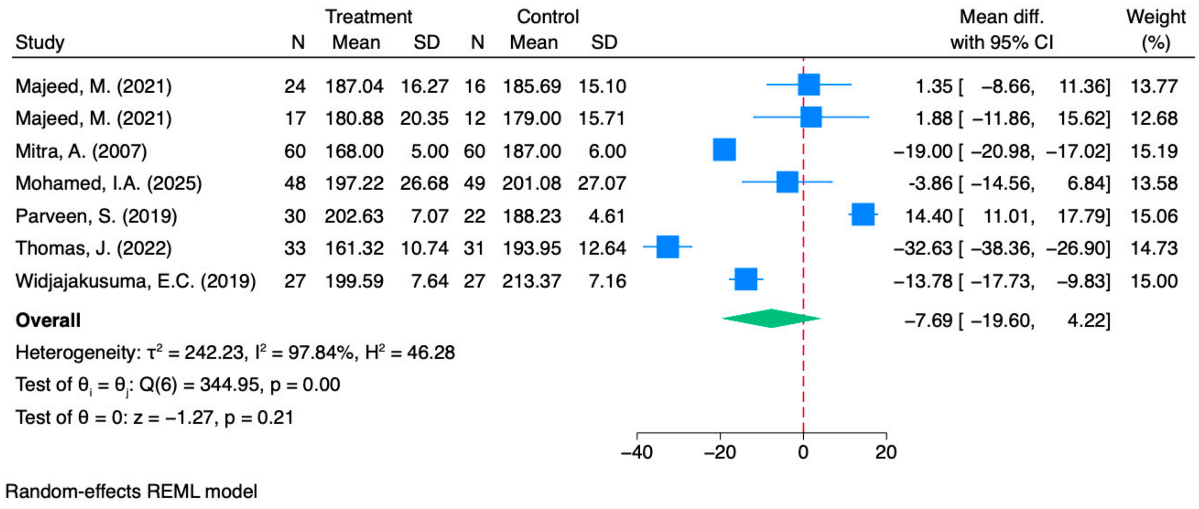

Figure S4. Forest plot of Myrtaceae interventions versus control for total cholesterol (mg/dL). Random-effects meta-analyses were performed using REML. Pooled effects are expressed as MD with 95% CI [27-29,31,33,34]. Blue squares, individual study MDs, with square size proportional to study weight; horizontal lines, 95% CIs; green diamond, pooled MD; red dashed vertical line, line of no effect.

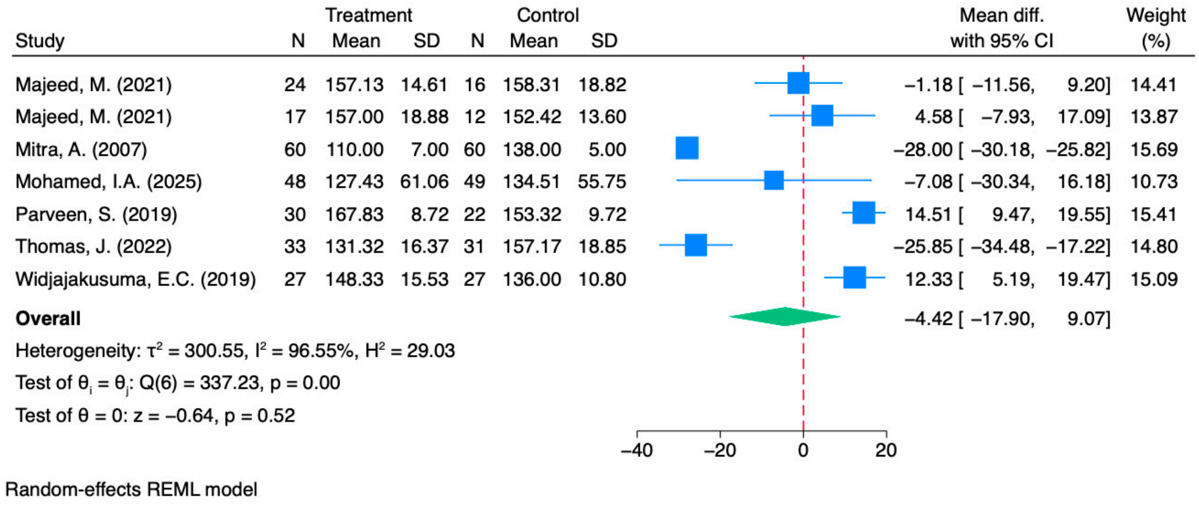

Figure S5. Forest plot of Myrtaceae interventions versus control for triglycerides (mg/dL). Random-effects meta-analyses were performed using REML. Pooled effects are expressed as MD with 95% CI [27-29,31,33,34]. Blue squares, individual study MDs, with square size proportional to study weight; horizontal lines, 95% CIs; green diamond, pooled MD; red dashed vertical line, line of no effect.

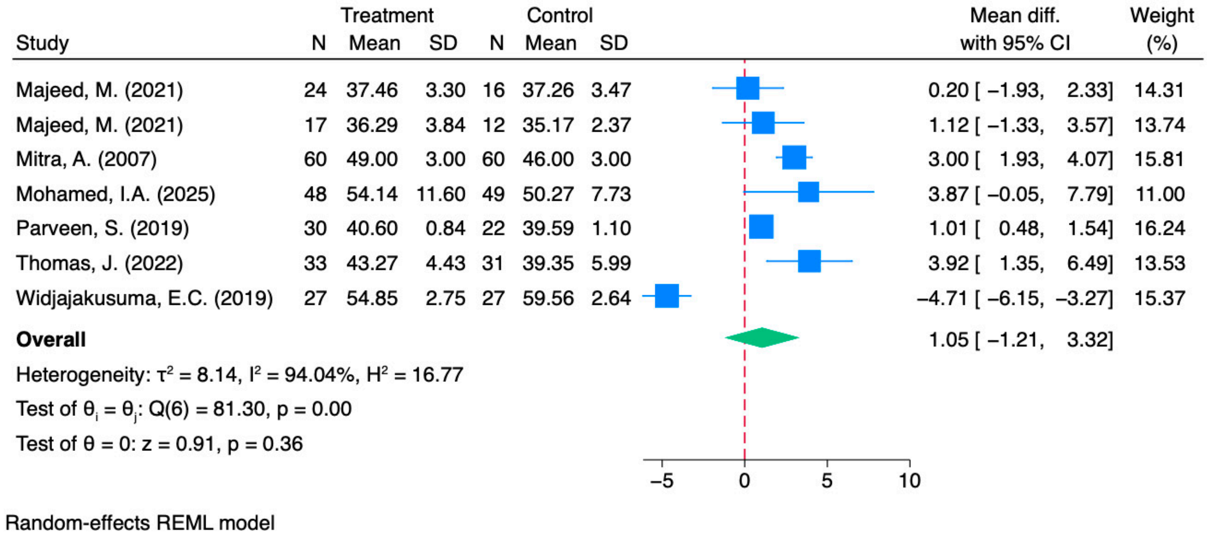

Figure S6. Forest plot of Myrtaceae interventions versus control for HDL (mg/dL). Random-effects meta-analyses were performed using REML. Pooled effects are expressed as MD with 95% CI [27-29,31,33,34]. Blue squares, individual study MDs, with square size proportional to study weight; horizontal lines, 95% CIs; green diamond, pooled MD; red dashed vertical line, line of no effect.

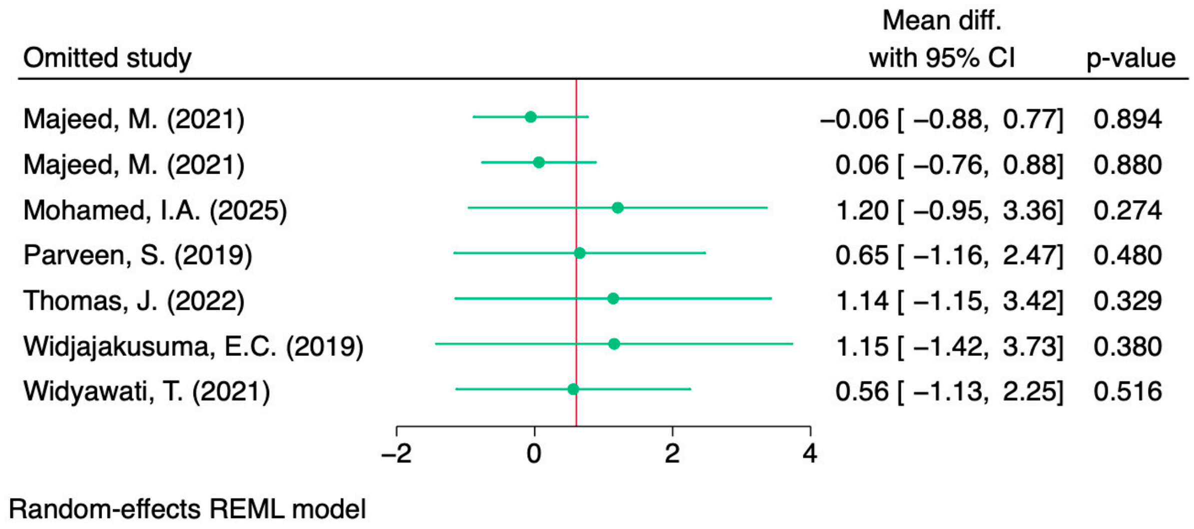

Figure S7. Leave-one-out sensitivity analysis for AST. Influence analysis showing the pooled MD after omitting each study sequentially [27,29,31,33-35]. Green circles and horizontal lines, omission-specific pooled MDs and 95% CIs; red vertical line, overall pooled MD from all included studies.

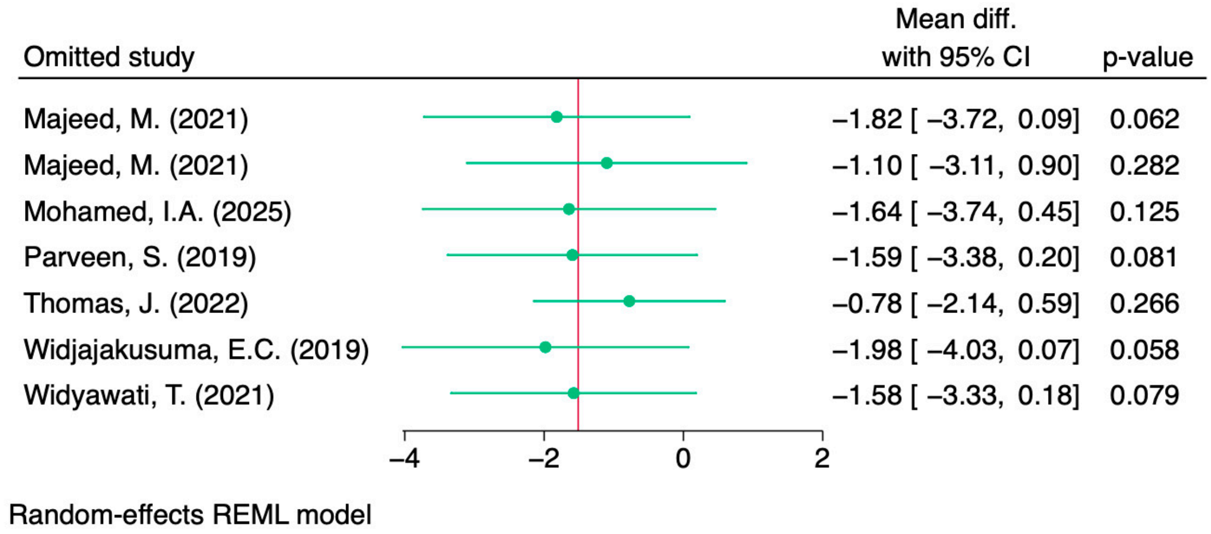

Figure S8. Leave-one-out sensitivity analysis for ALT. Influence analysis showing the pooled MD after omitting each study sequentially [27,29,31,33-35]. Green circles and horizontal lines, omission-specific pooled MDs and 95% CIs; red vertical line, overall pooled MD from all included studies.

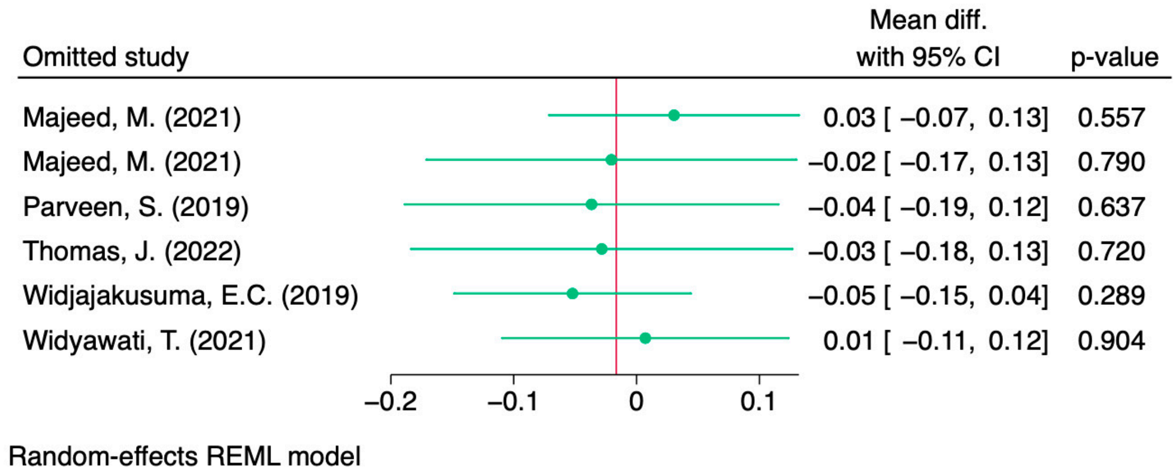

Figure S9. Leave-one-out sensitivity analysis for creatinine. Influence analysis showing the pooled MD after omitting each study sequentially [27,31,33-35]. Green circles and horizontal lines, omission-specific pooled MDs and 95% CIs; red vertical line, overall pooled MD from all included studies.
