# Supplementary material for: Efficacy and Safety of Syzygium cumini and Related Myrtaceae Interventions for Dysglycemia: A Systematic Review and Meta-Analysis of Randomized Controlled Trials
Source: Foods. 2026 Jul 1;15(13):2332. doi: 10.3390/foods15132332 (PMC13360929; doi:10.3390/foods15132332)
Supplement: Supplementary file 1 [file foods-15-02332-s001.zip › Table S1_search strategy.pdf]

Table S1. Search strategies

1. **PubMed** (Search date 3/9/2025)

Search number	Search Details	Results
1	"Syzygium cumini"[Title/Abstract]	424
2	"Eugenia jambolana"[Title/Abstract]	148
3	"Syzygium jambolanum"[Title/Abstract]	23
4	"jamun"[Title/Abstract]	138
5	"jambul"[Title/Abstract]	8
6	"jambolan"[Title/Abstract]	47
7	"Java plum"[Title/Abstract]	17
8	"Indian blackberry"[Title/Abstract]	11
9	"Syzygium"[Title/Abstract]	1,672
10	"Psidium guajava"[Title/Abstract]	1,045
11	"Eugenia uniflora"[Title/Abstract]	215
12	"Myrtus communis"[Title/Abstract]	413
13	"Pimenta dioica"[Title/Abstract]	68
14	"Eucalyptus globulus"[Title/Abstract]	999
15	"Myrtaceae"[Title/Abstract]	1,629
16	"Syzygium"[MeSH Terms]	1,303
17	"Myrtaceae"[MeSH Terms]	7,364
18	"Syzygium cumini"[Title/Abstract] OR "Eugenia jambolana"[Title/Abstract] OR "Syzygium jambolanum"[Title/Abstract] OR "jamun"[Title/Abstract] OR "jambul"[Title/Abstract] OR "jambolan"[Title/Abstract] OR "Java plum"[Title/Abstract] OR "Indian blackberry"[Title/Abstract] OR "Syzygium"[Title/Abstract] OR "Psidium guajava"[Title/Abstract] OR "Eugenia uniflora"[Title/Abstract] OR "Myrtus communis"[Title/Abstract] OR "Pimenta dioica"[Title/Abstract] OR "Eucalyptus globulus"[Title/Abstract] OR "Myrtaceae"[Title/Abstract] OR "Syzygium"[MeSH Terms] OR "Myrtaceae"[MeSH Terms]	10,150
19	"Diabetes mellitus"[MeSH Terms]	553,821
20	"Diabetes mellitus, type 2"[MeSH Terms]	192,314
21	"Diabetes mellitus, type 1"[MeSH Terms]	92,018
22	"Metabolic syndrome"[MeSH Terms]	41,936
23	"Hyperglycemia"[MeSH Terms]	43,545
24	"hyperglyc*"[Title/Abstract]	86,565
25	"Insulin resist*"[Title/Abstract]	115,802
26	"T2DM"[Title/Abstract]	42,810
27	"T1DM"[Title/Abstract]	7,938
28	"Diabetes"[Title/Abstract]	757,232
29	"Diabetes Mellitus"[MeSH Terms] OR "diabetes mellitus, type 2"[MeSH Terms] OR "diabetes mellitus, type 1"[MeSH Terms] OR "Metabolic Syndrome"[MeSH Terms] OR "Hyperglycemia"[MeSH Terms] OR "hyperglyc*"[Title/Abstract] OR "insulin resist*"[Title/Abstract] OR "T2DM"[Title/Abstract] OR "T1DM"[Title/Abstract] OR "Diabetes"[Title/Abstract]	1,002,326
30	("Syzygium cumini"[Title/Abstract] OR "Eugenia jambolana"[Title/Abstract] OR "Syzygium jambolanum"[Title/Abstract] OR "jamun"[Title/Abstract] OR "jambul"[Title/Abstract] OR "jambolan"[Title/Abstract] OR "Java plum"[Title/Abstract] OR "Indian blackberry"[Title/Abstract] OR "Syzygium"[Title/Abstract] OR "Psidium guajava"[Title/Abstract] OR "Eugenia uniflora"[Title/Abstract] OR "Myrtus communis"[Title/Abstract] OR "Pimenta dioica"[Title/Abstract] OR "Eucalyptus globulus"[Title/Abstract] OR "Myrtaceae"[Title/Abstract] OR "Syzygium"[MeSH Terms] OR "Myrtaceae"[MeSH Terms]) AND ("Diabetes Mellitus"[MeSH Terms] OR "diabetes mellitus, type	520

Search number	Search Details	Results
	2"[MeSH Terms] OR "diabetes mellitus, type 1"[MeSH Terms] OR "Metabolic Syndrome"[MeSH Terms] OR "Hyperglycemia"[MeSH Terms] OR "hyperglyc*"[Title/Abstract] OR "insulin resist*"[Title/Abstract] OR "T2DM"[Title/Abstract] OR "T1DM"[Title/Abstract] OR "Diabetes"[Title/Abstract])	

2. **Scopus** (Search date 3/9/2025)

Search number	Search Details	Results
1	TITLE-ABS-KEY ("Syzygium cumini")	2,224
2	TITLE-ABS-KEY ("Eugenia jambolana")	340
3	TITLE-ABS-KEY ("Syzygium jambolanum")	58
4	TITLE-ABS-KEY (jamun)	569
5	TITLE-ABS-KEY (jambul)	44
6	TITLE-ABS-KEY (jambolan)	142
7	TITLE-ABS-KEY ("Java plum")	87
8	TITLE-ABS-KEY ("Indian blackberry")	49
9	TITLE-ABS-KEY (duhat)	9
10	TITLE-ABS-KEY (Syzygium)	8,144
11	TITLE-ABS-KEY ("Psidium guajava")	4,269
12	TITLE-ABS-KEY ("Eugenia uniflora")	731
13	TITLE-ABS-KEY ("Myrtus communis")	1,319
14	TITLE-ABS-KEY ("Pimenta dioica")	282
15	TITLE-ABS-KEY ("Eucalyptus globulus")	4,914
16	TITLE-ABS-KEY (Myrtaceae)	8,355
17	(TITLE-ABS-KEY ("Syzygium cumini")) OR (TITLE-ABS-KEY ("Eugenia jambolana")) OR (TITLE-ABS-KEY ("Syzygium jambolanum")) OR (TITLE-ABS-KEY (jamun)) OR (TITLE-ABS-KEY (jambul)) OR (TITLE-ABS-KEY (jambolan)) OR (TITLE-ABS-KEY ("Java plum")) OR (TITLE-ABS-KEY ("Indian blackberry")) OR (TITLE-ABS-KEY (duhat)) OR (TITLE-ABS-KEY (Syzygium)) OR (TITLE-ABS-KEY ("Psidium guajava")) OR (TITLE-ABS-KEY ("Eugenia uniflora")) OR (TITLE-ABS-KEY ("Myrtus communis")) OR (TITLE-ABS-KEY ("Pimento dioica")) OR (TITLE-ABS-KEY ("Eucalyptus globulus")) OR (TITLE-ABS-KEY (Myrtaceae))	25,901
18	TITLE-ABS-KEY (prediabet*)	19,036
19	TITLE-ABS-KEY ("impaired glucose")	41,580
20	TITLE-ABS-KEY ("metabolic syndrome")	117,355
21	TITLE-ABS-KEY (hyperglyc*)	162,751
22	TITLE-ABS-KEY ("insulin resist*")	185,674
23	TITLE-ABS-KEY (T2DM)	49,677
24	TITLE-ABS-KEY ("type 2 diabetes")	238,497
25	TITLE-ABS-KEY ("type 1 diabetes")	70,655
26	TITLE-ABS-KEY (T1DM)	9,370
27	(TITLE-ABS-KEY (prediabet*)) OR (TITLE-ABS-KEY ("impaired glucose")) OR (TITLE-ABS-KEY ("metabolic syndrome")) OR (TITLE-ABS-KEY (hyperglyc*)) OR (TITLE-ABS-KEY ("insulin resist*")) OR (TITLE-ABS-KEY (T2DM)) OR (TITLE-ABS-KEY ("type 2 diabetes")) OR (TITLE-ABS-KEY ("type 1 diabetes")) OR (TITLE-ABS-KEY (T1DM))	642,280
28	(TITLE-ABS-KEY (prediabet*)) OR (TITLE-ABS-KEY ("impaired glucose")) OR (TITLE-ABS-KEY ("metabolic syndrome")) OR (TITLE-ABS-KEY (hyperglyc*)) OR (TITLE-ABS-KEY ("insulin resist*")) OR (TITLE-ABS-KEY (	567

Search number	Search Details	Results
	T2DM)) OR (TITLE-ABS-KEY ("type 2 diabetes")) OR (TITLE-ABS-KEY ("type 1 diabetes")) OR (TITLE-ABS-KEY (T1DM))) AND ((TITLE-ABS-KEY ("Syzygium cumini")) OR (TITLE-ABS-KEY ("Eugenia jambolana")) OR (TITLE-ABS-KEY ("Syzygium jambolanum")) OR (TITLE-ABS-KEY (jamun)) OR (TITLE-ABS-KEY (jambul)) OR (TITLE-ABS-KEY (jambolan)) OR (TITLE-ABS-KEY ("Java plum")) OR (TITLE-ABS-KEY ("Indian blackberry")) OR (TITLE-ABS-KEY (duhat)) OR (TITLE-ABS-KEY (Syzygium)) OR (TITLE-ABS-KEY ("Psidium guajava")) OR (TITLE-ABS-KEY ("Eugenia uniflora")) OR (TITLE-ABS-KEY ("Myrtus communis")) OR (TITLE-ABS-KEY ("Pimenta dioica")) OR (TITLE-ABS-KEY ("Eucalyptus globulus")) OR (TITLE-ABS-KEY (Myrtaceae)))	

3. **Embase** (Search date 3/9/2025)

Search number	Search Details	Results
1	exp Syzygium cumini/ or exp Syzygium cumini extract/ or 'Syzygium cumini'.mp.	1,539
2	'Eugenia jambolana'.mp.	271
3	'Syzygium jambolanum'.mp.	35
4	Syzygium.mp. or exp Syzygium malaccense/ or exp Syzygium/ or exp Syzygium samarangense/ or exp Syzygium polyanthum/	5,540
5	exp Myrtus communis extract/ or 'Myrtus communis'.mp.	741
6	Myrtaceae.mp. or exp Myrtaceae/	17,298
7	1 or 2 or 3 or 4 or 5 or 6	18,360
8	exp non insulin dependent diabetes mellitus/ or 'type 2 diabetes mellitus'.mp.	442,150
9	exp insulin dependent diabetes mellitus/ or 'type 1 diabetes mellitus'.mp.	185,260
10	hyperglycemia.mp. or exp hyperglycemia/ or exp postprandial hyperglycemia/	171,827
11	exp impaired glucose tolerance/ or prediabet*.mp.	52,080
12	Diabetes.mp. or exp diabetes mellitus/	1,726,468
13	8 or 9 or 10 or 11 or 12	1,784,552
14	HbA1c.mp. or exp hemoglobin A1c/	203,190
15	exp glucose blood level/ or fasting plasma glucose.mp. or exp glucose/	816,943
16	exp insulin blood level/ or exp insulin/ or "fasting insulin".mp.	477,928
17	"lipid profile".mp.	57,274
18	"adverse events".mp. or exp adverse event/	1,598,124
19	"blood pressure".mp. or exp blood pressure/	1,085,930
20	14 or 15 or 16 or 17 or 18 or 19	3,630,322
21	7 and 13 and 20	682

4. **MEDLINE** (Search date 3/9/2025)

Search number	Search Details	Results
1	"Syzygium cumini".mp. or exp Syzygium/	1,536
2	"Eugenia jambolana".mp.	150
3	"Syzygium jambolanum".mp.	23
4	Myrtaceae.mp. or exp Myrtaceae/	8,065
5	1 or 2 or 3 or 4	8,337
6	"Diabetes Mellitus".mp. or exp Diabetes Mellitus/	665,369
7	exp Diabetes Mellitus, Type 2/ or T2DM.mp.	206,002
8	exp Diabetes Mellitus, Type 1/ or T1DM.mp.	94,447

9	"Metabolic Syndrome".mp. or exp Metabolic Syndrome/	79,366
10	Hyperglycemia.mp. or exp Hyperglycemia/	85,716
11	exp Prediabetic State/ or prediabet*.mp.	17,359
12	6 or 7 or 8 or 9 or 10 or 11	764,333
13	"fasting plasma glucose".mp.	17,056
14	exp Glycated Hemoglobin/ or exp Blood Glucose/ or HbA1c.mp.	244,988
15	"fasting insulin".mp.	10,292
16	exp Dyslipidemias/ or "lipid profile".mp. or exp Cardiovascular Diseases/	2,961,873
17	exp Cholesterol, HDL/ or exp Cholesterol/ or exp Cholesterol, LDL/ or exp Cholesterol, VLDL/ or Cholesterol.mp.	352,703
18	exp Triglycerides/ or tryglyceride.mp.	89,416
19	13 or 14 or 15 or 16 or 17 or 18	3,394,149
20	5 and 12 and 19	177

5. **Web of Science** (Search date 3/9/2025)

Search number	Search Details	Results
1	"Syzygium cumini" (Topic)	1,195
2	"Eugenia jambolana" (Topic)	400
3	"Syzygium jambolanum" (Topic)	38
4	Syzygium (Topic)	4,578
5	"Psidium guajava" (Topic)	3,501
6	"Eugenia uniflora" (Topic)	560
7	"Myrtus communis" (Topic)	1,152
8	"Pimenta dioica" (Topic)	183
9	"Eucalyptus globulus" (Topic)	4,883
10	Myrtaceae (Topic)	6,493
11	#1 OR #2 OR #3 OR #4 OR #5 OR #6 OR #7 OR #8 OR #9 OR #10	19,808
12	diabetes (Topic)	905,721
13	prediabet* (Topic)	15,572
14	"impaired glucose" (Topic)	25,822
15	"metabolic syndrome" (Topic)	134,714
16	hyperglyc* (Topic)	112,739
17	"insulin resist*" (Topic)	212,501
18	#12 OR #13 OR #14 OR #15 OR #16 OR #17	1,128,672
19	HbA1c (Topic)	50,316
20	"fasting plasma glucose" (Topic)	17,930
21	"fasting blood glucose" (Topic)	20,128
22	"blood glucose level" (Topic)	11,808
23	"fasting insulin" (Topic)	10,398
24	"lipid profile" (Topic)	41,528
25	cholesterol (Topic)	398,855
26	triglyceride (Topic)	177,026
27	"adverse event" (Topic)	47,725
28	#19 OR #20 OR #21 OR #22 OR #23 OR #24 OR #25 OR #26 OR #27	642,792
29	#11 AND #18 AND #28	719
