# Supplementary material for: Efficacy and Safety of Syzygium cumini and Related Myrtaceae Interventions for Dysglycemia: A Systematic Review and Meta-Analysis of Randomized Controlled Trials
Source: Foods. 2026 Jul 1;15(13):2332. doi: 10.3390/foods15132332 (PMC13360929; doi:10.3390/foods15132332)
Supplement: Supplementary file 1 [file foods-15-02332-s001.zip › Table S2-S4_subgroup analysis.pdf]

Table S2. Subgroup analysis of glycemic outcomes

Subgroup categories	FPG			PPG			HbA1c		
	Mean diff (95% CI)	I^2	*P*	Mean diff (95% CI)	I^2	*P*	Mean diff (95% CI)	I^2	*P*
**Publication period**									
2001-2010	-15.938 (-18.092 to -13.785)	0.00	0.085	6.000 (-51.545 to 63.545)	-	0.264	-0.198 (-0.289 to -0.107)	0.00	0.499
2011-2020	-24.119 (-45.337 to -2.902)	98.87		-24.865 (-40.811 to -8.918)	96.00		-1.212 (-2.910 to 0.486)	98.96	
2021-2025	-7.872 (-15.222 to -0.522)	93.72		-4.010 (-28.087 to 20.067)	97.78		-0.226 (-0.517 to 0.065)	95.42	
**Participant**									
Prediabetes	-6.296 (-13.083 to 0.490)	95.67	0.053	-14.588 (-28.960 to -0.216)	95.99	0.781	-0.327 (-0.696 to 0.042)	96.83	0.648
T2DM	-20.478 (-33.114 to -7.843)	97.50		-10.445 (-35.944 to 15.054)	97.46		-0.538 (-1.365 to 0.289)	99.23	
**Plant species**									
Other Myrtaceae	-10.622 (-19.660 to -1.584)	92.86	0.493	-27.230 (-36.159 to -18.301)	43.03	0.077	-0.281 (-0.802 to 0.240)	98.59	0.577
S.cumini	-15.702 (-27.051 to -4.353)	98.82		-8.891 (-27.141 to 9.358)	98.25		-0.532 (-1.244 to 0.180)	98.96	
**Plant part**									
Fruit	-1.939 (-4.158 to 0.280)	0.00	0.008	-2.209 (-6.199 to 1.781)	0.00	0.000	-0.035 (-0.108 to 0.039)	0.01	0.408
Leaf	-9.403 (-15.663 to -3.143)	0.00		-22.180 (-31.011 to -13.349)	-		-0.030 (-0.147 to 0.087)	-	
Others	-19.592 (-36.422 to -2.761)	98.61		-31.807 (-38.084 to -25.529)	30.28		-0.876 (-1.926 to 0.174)	99.50	
Seed	-20.917 (-40.475 to -1.360)	97.36		-0.175 (-46.021 to 45.672)	98.43		-0.320 (-1.169 to 0.530)	72.42	
**Component**									
Mixed	-7.831 (-14.155 to -1.508)	89.98	0.110	-1.758 (-21.596 to 18.080)	95.66	0.051	-0.112 (-0.207 to -0.016)	50.86	0.113
Single	-21.468 (-36.925 to -6.011)	99.06		-27.160 (-43.201 to -11.118)	96.74		-1.102 (-2.324 to 0.120)	99.54	
**Comparator**									
Metformin	-6.709 (-21.010 to 7.591)	93.77	0.542	11.443 (-16.889 to 39.775)	97.38	0.020	-0.054 (-0.149 to 0.040)	0.01	0.053

Subgroup categories	FPG			PPG			HbA1c		
	Mean diff (95% CI)	I^2	*P*	Mean diff (95% CI)	I^2	*P*	Mean diff (95% CI)	I^2	*P*
Others	-12.868 (-24.988 to -0.749)	92.70		-29.533 (-36.318 to -22.748)	10.01		-0.474 (-0.845 to -0.103)	96.39	
Placebo	-17.590 (-30.517 to -4.663)	98.92		-23.148 (-38.465 to -7.832)	94.65		-0.733 (-1.864 to 0.397)	99.29	
**Treatment duration**									
0–8 weeks	-9.403 (-15.663 to -3.143)	0.00	0.299	-22.180 (-31.011 to -13.349)	5.18	0.572	-0.030 (-0.147 to 0.087)	-	0.093
17–24 weeks	-10.319 (-26.892 to 6.255)	98.22		-14.419 (-30.468 to 1.630)	93.82		-0.568 (-1.510 to 0.374)	99.34	
9–16 weeks	-19.169 (-29.871 to -8.466)	98.68		-8.305 (-43.599 to 26.990)	98.99		-0.444 (-0.852 to -0.036)	95.76	

Abbreviations: FPG, fasting plasma glucose; PPG, postprandial glucose; HbA1c, glycated hemoglobin; CI, confidence interval; I^2, heterogeneity within subgroup. *P* values represent the test for subgroup differences between categories. A dash (–) indicates not applicable or not estimable because only one study was available in that subgroup.

Table S3. Subgroup analysis of lipid outcomes

Subgroup categories	Total cholesterol			Triglycerides			HDL		
	Mean diff (95% CI)	I^2	*P*	Mean diff (95% CI)	I^2	*P*	Mean diff (95% CI)	I^2	*P*
**Publication period**									
2001-2010	-19.000 (-20.976 to -17.024)	-	0.200	-28.000 (-30.177 to -25.823)	-	0.000	3.000 (1.926 to 4.074)	-	0.195
2011-2020	0.329 (-27.287 to 27.945)	99.11		13.785 (9.671 to 17.899)	0.00		-1.809 (-7.414 to 3.796)	98.13	
2021-2025	-8.909 (-25.669 to 7.851)	91.95		-7.842 (-22.367 to 6.683)	82.07		2.011 (0.098 to 3.925)	51.64	
**Participant**									
Prediabetes	-5.160 (-25.320 to 15.000)	97.14	0.597	-3.134 (-21.621 to 15.353)	92.71	0.858	1.988 (0.414 to 3.561)	55.45	0.305
T2DM	-11.382 (-22.539 to -0.226)	95.26		-5.877 (-29.513 to 17.759)	97.70		-0.494 (-4.973 to 3.984)	96.34	
**Plant species**									
Other Myrtaceae	-17.150 (-33.481 to -0.819)	95.26	0.137	-6.742 (-30.294 to 16.811)	85.26	0.797	0.857 (-4.930 to 6.643)	93.82	0.837
S.cumini	-0.545 (-15.153 to 14.064)	97.58		-2.812 (-21.343 to 15.720)	97.57		1.478 (0.223 to 2.732)	73.25	

Subgroup categories	Total cholesterol			Triglycerides			HDL		
	**Mean diff (95% CI)**	***I*²**	***P***	**Mean diff (95% CI)**	***I*²**	***P***	**Mean diff (95% CI)**	***I*²**	***P***
**Plant part**									
Fruit	-0.429 (-6.881 to 6.024)	0.00	0.239	0.298 (-7.256 to 7.851)	0.00	0.566	1.068 (-0.420 to 2.557)	0.00	0.880
Others	-12.689 (-32.031 to 6.652)	99.22		-6.753 (-29.643 to 16.137)	98.79		0.757 (-3.009 to 4.523)	97.72	
**Component**									
Mixed	-8.770 (-19.103 to 1.563)	93.89	0.923	-3.483 (-21.373 to 14.406)	96.22	0.879	-0.111 (-3.440 to 3.219)	93.89	0.208
Single	-7.333 (-34.502 to 19.835)	98.44		-5.855 (-30.636 to 18.927)	94.99		2.460 (0.239 to 4.682)	66.57	
**Comparator**									
Metformin	1.534 (-6.556 to 9.623)	0.00	0.003	1.167 (-6.819 to 9.153)	0.00	0.000	0.595 (-1.013 to 2.204)	0.00	0.029
Others	-25.538 (-38.884 to -12.192)	94.84		-27.872 (-29.982 to -25.761)	0.00		3.137 (2.146 to 4.127)	0.00	
Placebo	-0.949 (-17.727 to 15.828)	97.19		13.152 (9.101 to 17.203)	0.00		-0.152 (-5.015 to 4.710)	96.95	
**Treatment duration**									
0–8 weeks	-13.780 (-17.729 to -9.831)	-	0.002	12.330 (5.195 to 19.465)	-	0.029	-4.710 (-6.148 to -3.272)	-	0.000
17–24 weeks	-0.429 (-6.881 to 6.024)	0.00		0.298 (-7.256 to 7.851)	0.00		1.068 (-0.420 to 2.557)	0.00	
9–16 weeks	-12.354 (-39.704 to 14.997)	99.49		-13.067 (-40.245 to 14.110)	98.98		2.375 (0.675 to 4.076)	84.64	

Abbreviations: HDL, high-density lipoprotein cholesterol; CI, confidence interval; I^2, heterogeneity within subgroup. *P* values represent the test for subgroup differences between categories. A dash (–) indicates not applicable or not estimable because only one study was available in that subgroup.

Table S4. Subgroup analysis of safety outcomes

Subgroup categories	AST			ALT			Creatinine		
	Mean diff (95% CI)	I^2	*P*	Mean diff (95% CI)	I^2	*P*	Mean diff (95% CI)	I^2	*P*
**Publication period**									
2011-2020	0.008 (-0.916 to 0.931)	0.00	0.419	-0.273 (-1.248 to 0.703)	0.00	0.117	0.113 (-0.025 to 0.250)	91.22	0.030
2021-2025	1.228 (-1.583 to 4.039)	56.60		-2.115 (-4.203 to -0.026)	50.69		-0.087 (-0.205 to 0.031)	72.58	
**Participant**									
Prediabetes	-0.327 (-2.127 to 1.473)	0.00	0.287	-2.771 (-4.686 to -0.857)	36.11	0.019	0.009 (-0.029 to 0.047)	0.01	0.558
T2DM	2.219 (-2.111 to 6.550)	67.28		-0.218 (-1.163 to 0.727)	0.00		-0.082 (-0.385 to 0.221)	93.20	
**Plant species**									
Other Myrtaceae	-0.158 (-0.999 to 0.682)	0.00	0.011	-1.546 (-3.972 to 0.880)	72.83	0.919	0.007 (-0.227 to 0.241)	96.96	0.654
S.cumini	3.766 (0.874 to 6.658)	0.00		-1.330 (-4.677 to 2.016)	45.46		-0.055 (-0.192 to 0.083)	80.86	
**Plant part**									
Fruit	1.962 (-1.908 to 5.833)	68.42	0.563	-1.391 (-3.648 to 0.865)	40.05	0.148	-0.112 (-0.279 to 0.054)	71.98	0.350
Leaf	0.016 (-0.911 to 0.943)	0.00		-0.272 (-1.251 to 0.706)	0.00		-0.035 (-0.520 to 0.450)	85.61	
Others	-0.587 (-3.343 to 2.169)	0.00		-3.567 (-6.959 to -0.174)	12.91		0.014 (-0.026 to 0.053)	0.05	
**Component**									
Mixed	2.101 (-1.117 to 5.318)	67.78	0.129	-1.009 (-3.074 to 1.056)	58.91	0.598	-0.010 (-0.228 to 0.209)	93.60	0.865
Single	-0.808 (-2.747 to 1.131)	0.00		-2.018 (-5.155 to 1.118)	48.10		0.010 (-0.030 to 0.049)	0.03	
**Comparator**									
Metformin	4.092 (1.047 to 7.137)	0.00	0.029	-1.600 (-5.349 to 2.150)	64.47	0.003	-0.112 (-0.279 to 0.054)	71.98	0.429
Others	-0.720 (-3.608 to 2.168)	-		-4.130 (-6.167 to -2.093)	-		0.000 (-0.049 to 0.049)	-	

Subgroup categories	AST			ALT			Creatinine		
	**Mean diff (95% CI)**	***I*²**	***P***	**Mean diff (95% CI)**	***I*²**	***P***	**Mean diff (95% CI)**	***I*²**	***P***
Placebo	-0.098 (-0.973 to 0.776)	0.00		-0.300 (-1.224 to 0.624)	0.00		0.026 (-0.202 to 0.253)	95.18	
**Treatment duration**									
0–8 weeks	0.016 (-0.911 to 0.943)	0.00	0.563	-0.272 (-1.251 to 0.706)	0.00	0.148	-0.035 (-0.520 to 0.450)	85.61	0.350
17–24 weeks	1.962 (-1.908 to 5.833)	68.42		-1.391 (-3.648 to 0.865)	40.05		-0.112 (-0.279 to 0.054)	71.98	
9–16 weeks	-0.587 (-3.343 to 2.169)	0.00		-3.567 (-6.959 to -0.174)	12.91		0.014 (-0.026 to 0.053)	0.05	

Abbreviations: AST, aspartate aminotransferase; ALT, alanine aminotransferase; CI, confidence interval; I^2, heterogeneity within subgroup. *P* values represent the test for subgroup differences between categories. A dash (–) indicates not applicable or not estimable because only one study was available in that subgroup.
